# Supplementary figures and images for: The nutrient-sensing GCN2 signaling pathway is essential for circadian clock function by regulating histone acetylation under amino acid starvation
Source: eLife. 2023 Apr 21;12:e85241. doi: 10.7554/eLife.85241 (PMC10191625; doi:10.7554/eLife.85241)

WT

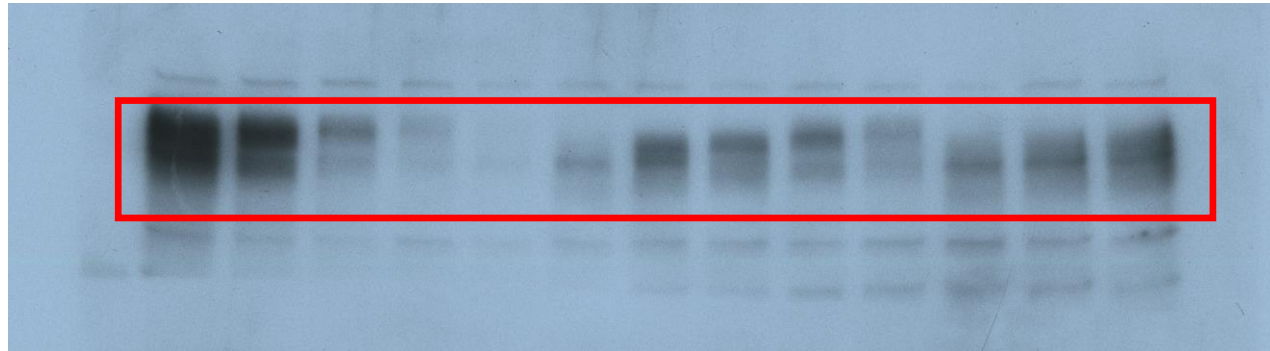

*cpc-3*<sup>ko</sup>

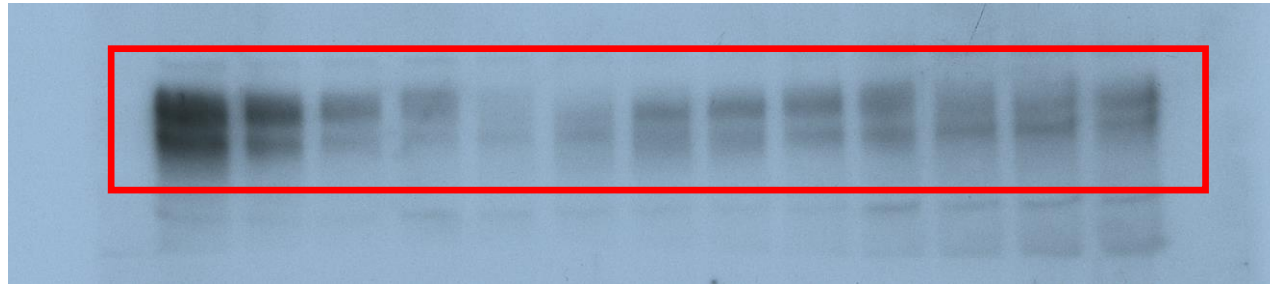

*cpc-1*<sup>ko</sup>

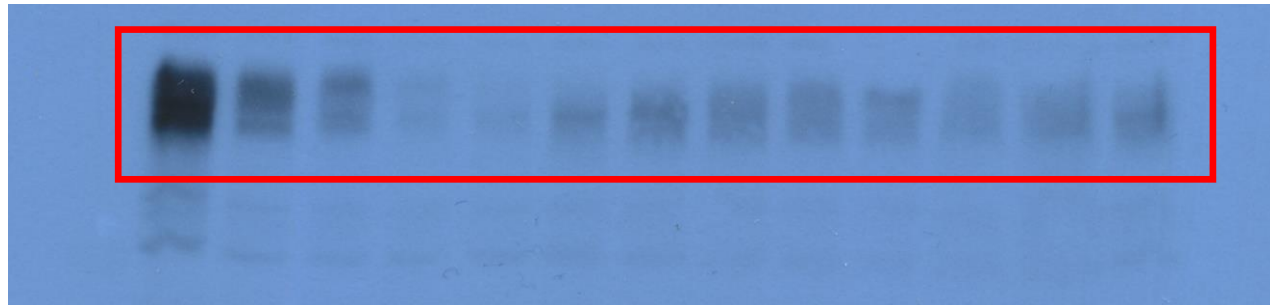

Supplement: Figure 1—source data 2. [file elife-85241-fig1-data2.zip › Figure 1-source data 2/Figure 1-source data 2-1.pdf]

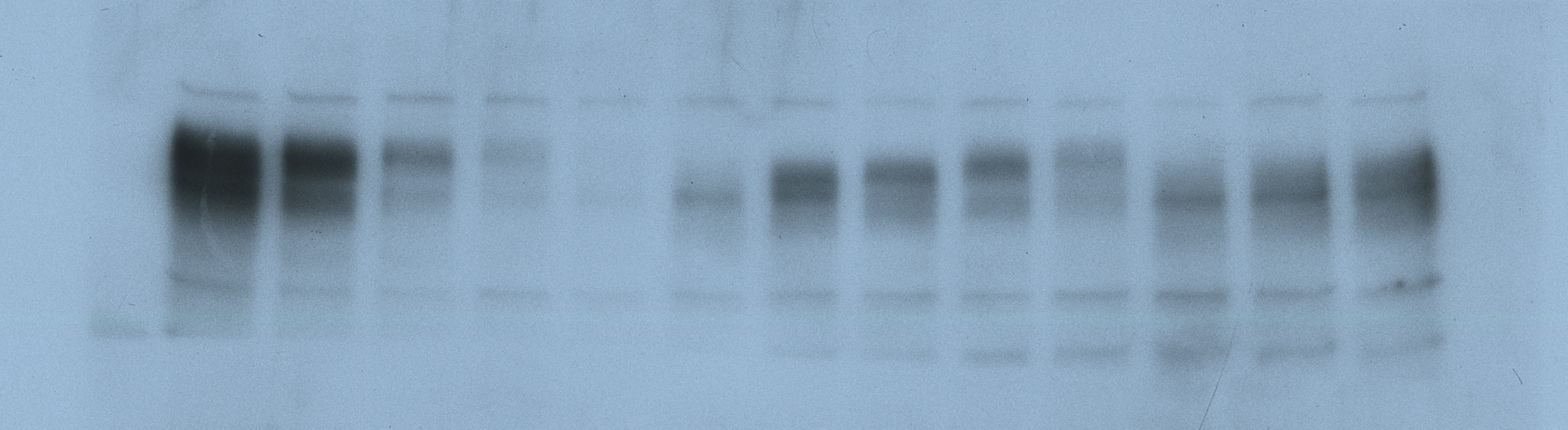

Supplement: Figure 1—source data 2. [file elife-85241-fig1-data2.zip › Figure 1-source data 2/Figure 1-source data 2-2.tif]

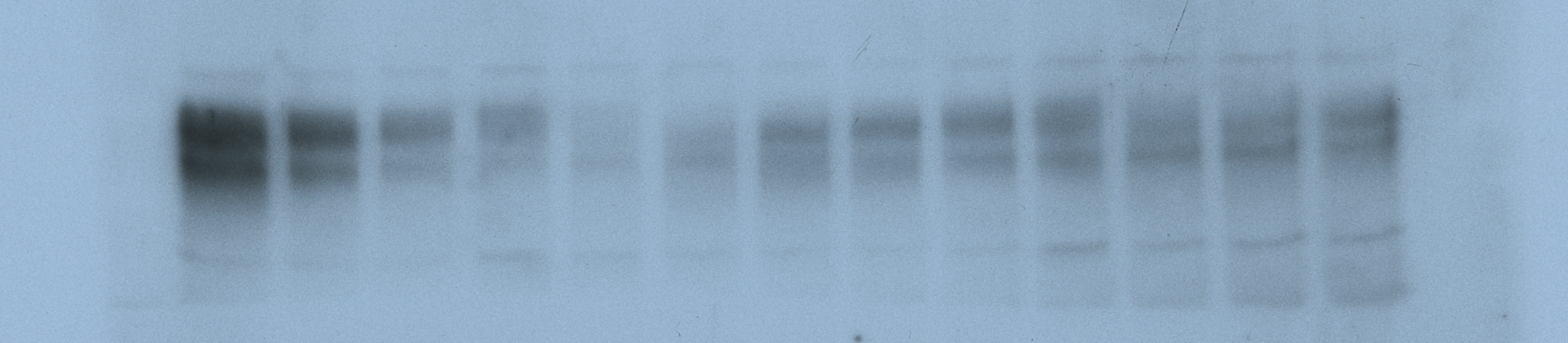

Supplement: Figure 1—source data 2. [file elife-85241-fig1-data2.zip › Figure 1-source data 2/Figure 1-source data 2-3.tif]

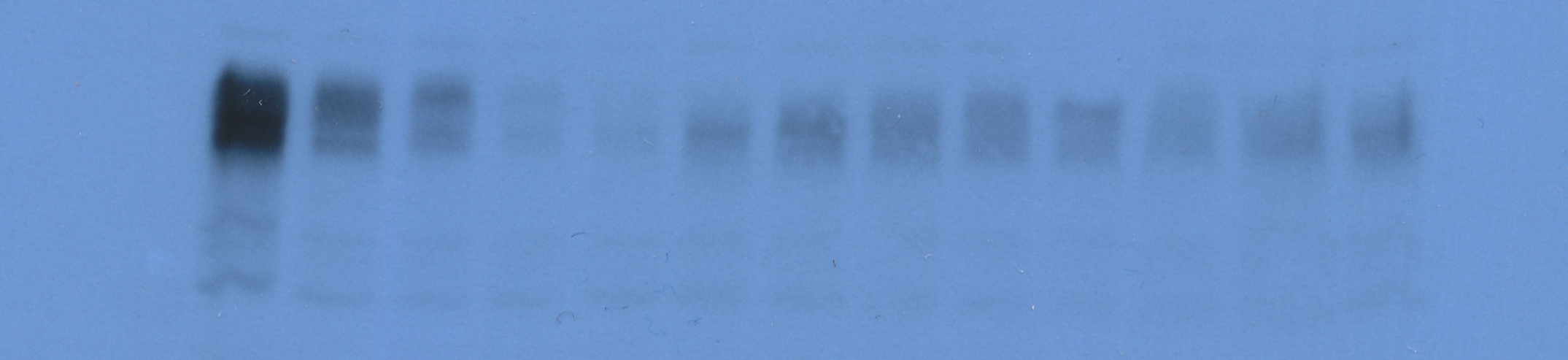

Supplement: Figure 1—source data 2. [file elife-85241-fig1-data2.zip › Figure 1-source data 2/Figure 1-source data 2-4.tif]

## Northern

## Gel

WT

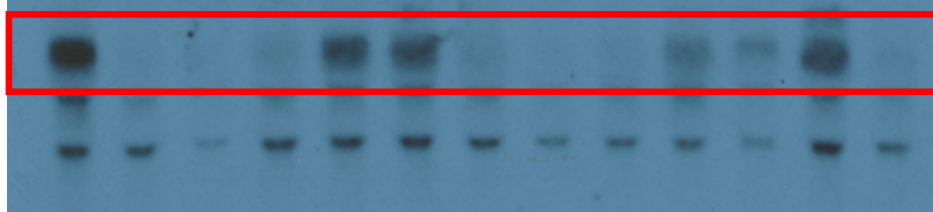

*cpc-3*<sup>ko</sup>

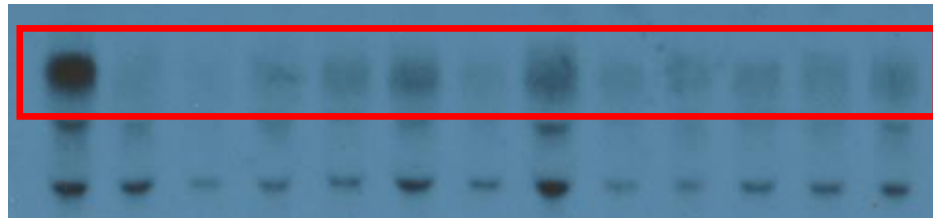

*cpc-1*<sup>ko</sup>

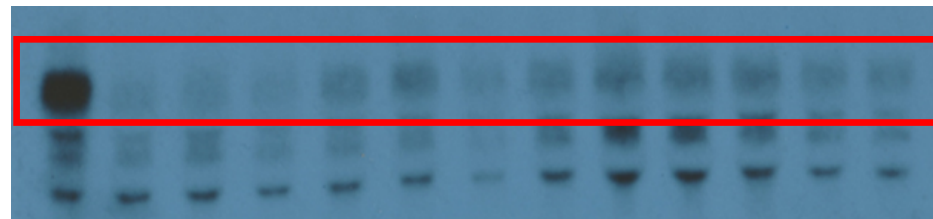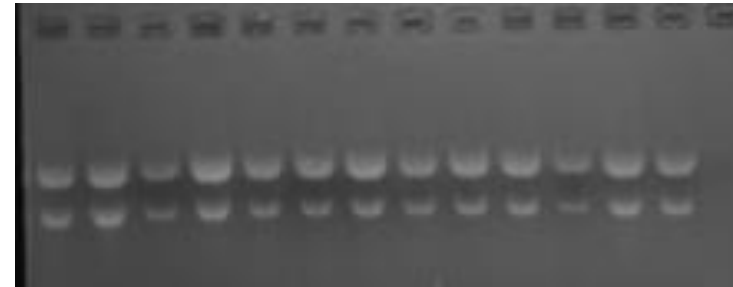

WT

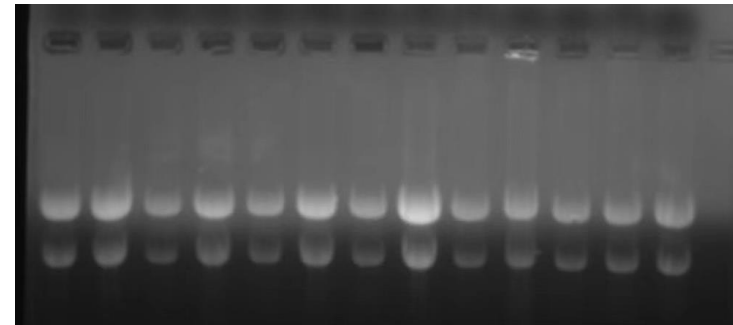

*cpc-3*<sup>ko</sup>

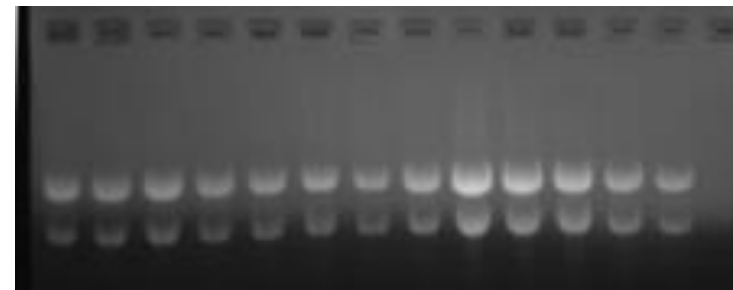

*cpc-1*<sup>ko</sup>

Supplement: Figure 1—source data 3. [file elife-85241-fig1-data3.zip › Figure 1-source data 3/Figure 1-source data 3-1.pdf]

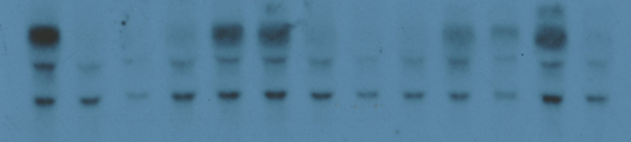

Supplement: Figure 1—source data 3. [file elife-85241-fig1-data3.zip › Figure 1-source data 3/Figure 1-source data 3-2.TIFF]

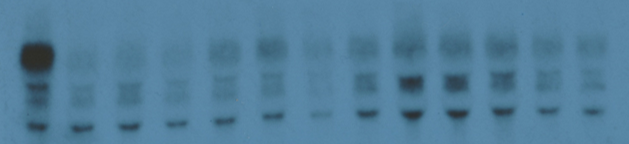

Supplement: Figure 1—source data 3. [file elife-85241-fig1-data3.zip › Figure 1-source data 3/Figure 1-source data 3-3.TIFF]

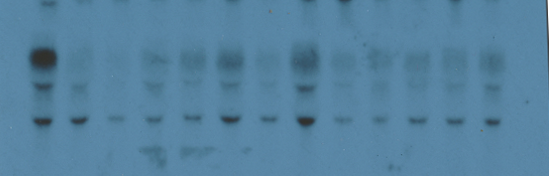

Supplement: Figure 1—source data 3. [file elife-85241-fig1-data3.zip › Figure 1-source data 3/Figure 1-source data 3-4.TIFF]

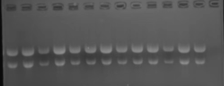

Supplement: Figure 1—source data 3. [file elife-85241-fig1-data3.zip › Figure 1-source data 3/Figure 1-source data 3-5.TIFF]

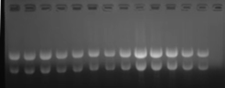

Supplement: Figure 1—source data 3. [file elife-85241-fig1-data3.zip › Figure 1-source data 3/Figure 1-source data 3-6.TIFF]

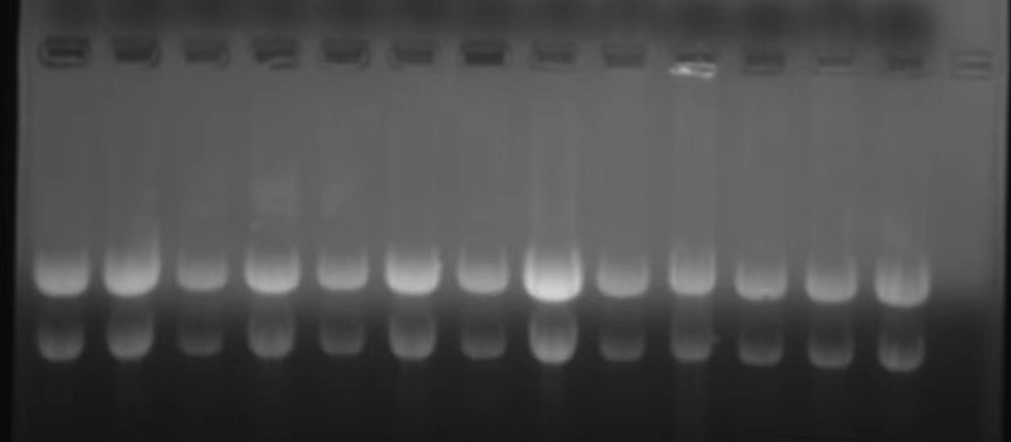

Supplement: Figure 1—source data 3. [file elife-85241-fig1-data3.zip › Figure 1-source data 3/Figure 1-source data 3-7.TIFF]

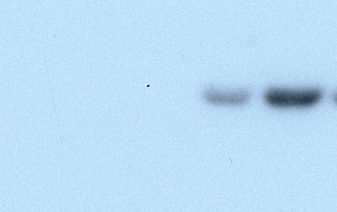

Supplement: Figure 1—figure supplement 1—source data 1. [file elife-85241-fig1-figsupp1-data1.zip › Figure 1-figure supplement 1-source data 1/Figure 1-figure supplement 1-source data 1-1.tif]

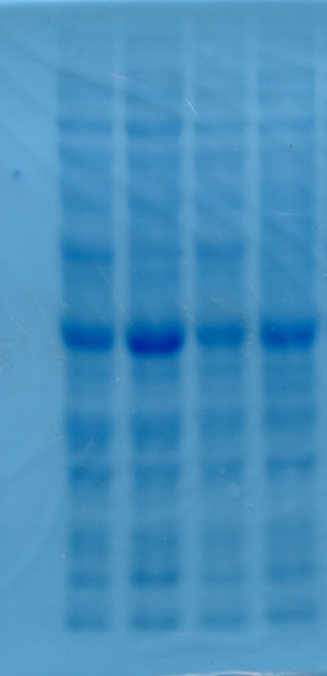

Supplement: Figure 1—figure supplement 1—source data 1. [file elife-85241-fig1-figsupp1-data1.zip › Figure 1-figure supplement 1-source data 1/Figure 1-figure supplement 1-source data 1-2.tif]

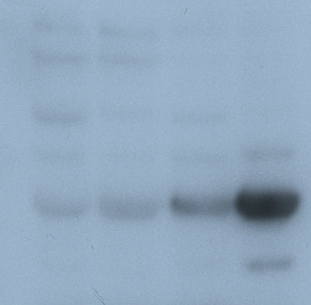

Supplement: Figure 1—figure supplement 1—source data 1. [file elife-85241-fig1-figsupp1-data1.zip › Figure 1-figure supplement 1-source data 1/Figure 1-figure supplement 1-source data 1-3.tif]

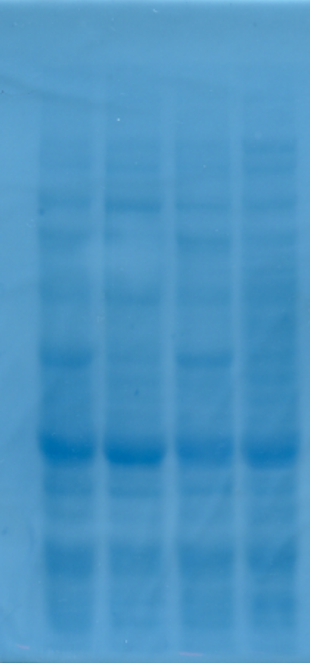

Supplement: Figure 1—figure supplement 1—source data 1. [file elife-85241-fig1-figsupp1-data1.zip › Figure 1-figure supplement 1-source data 1/Figure 1-figure supplement 1-source data 1-4.tif]

P-eIF2 $\alpha$

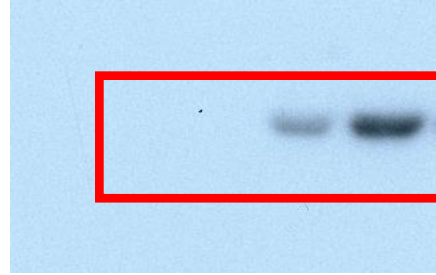

membrane

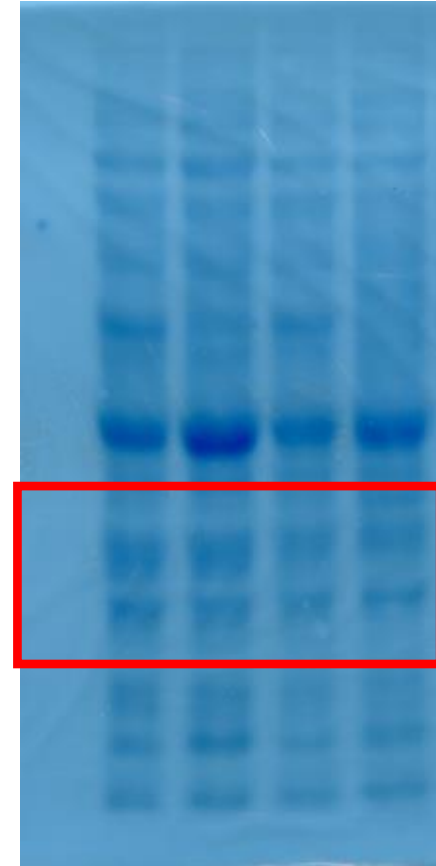

CPC-1

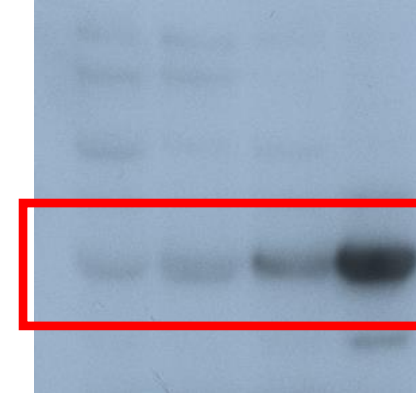

membrane

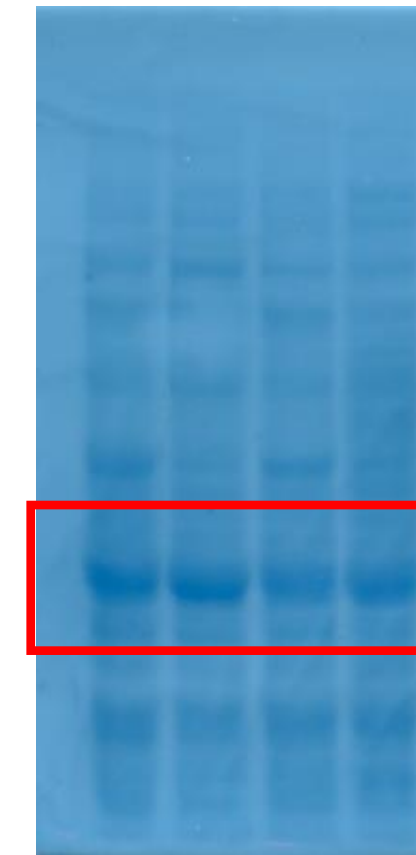

Supplement: Figure 1—figure supplement 1—source data 1. [file elife-85241-fig1-figsupp1-data1.zip › Figure 1-figure supplement 1-source data 1/Figure 1-figure supplement 1-source data 1.pdf]

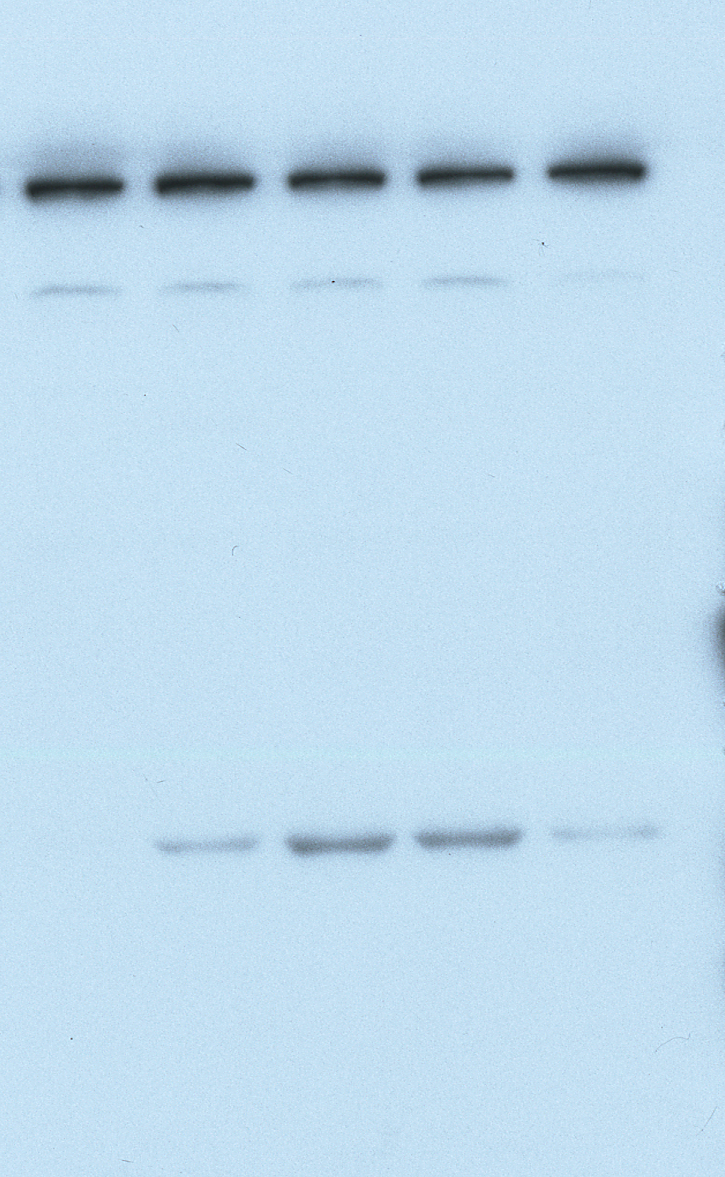

Supplement: Figure 1—figure supplement 1—source data 2. [file elife-85241-fig1-figsupp1-data2.zip › Figure 1-figure supplement 1-source data 2/Figure 1-figure supplement 1-source data 2-1.tif]

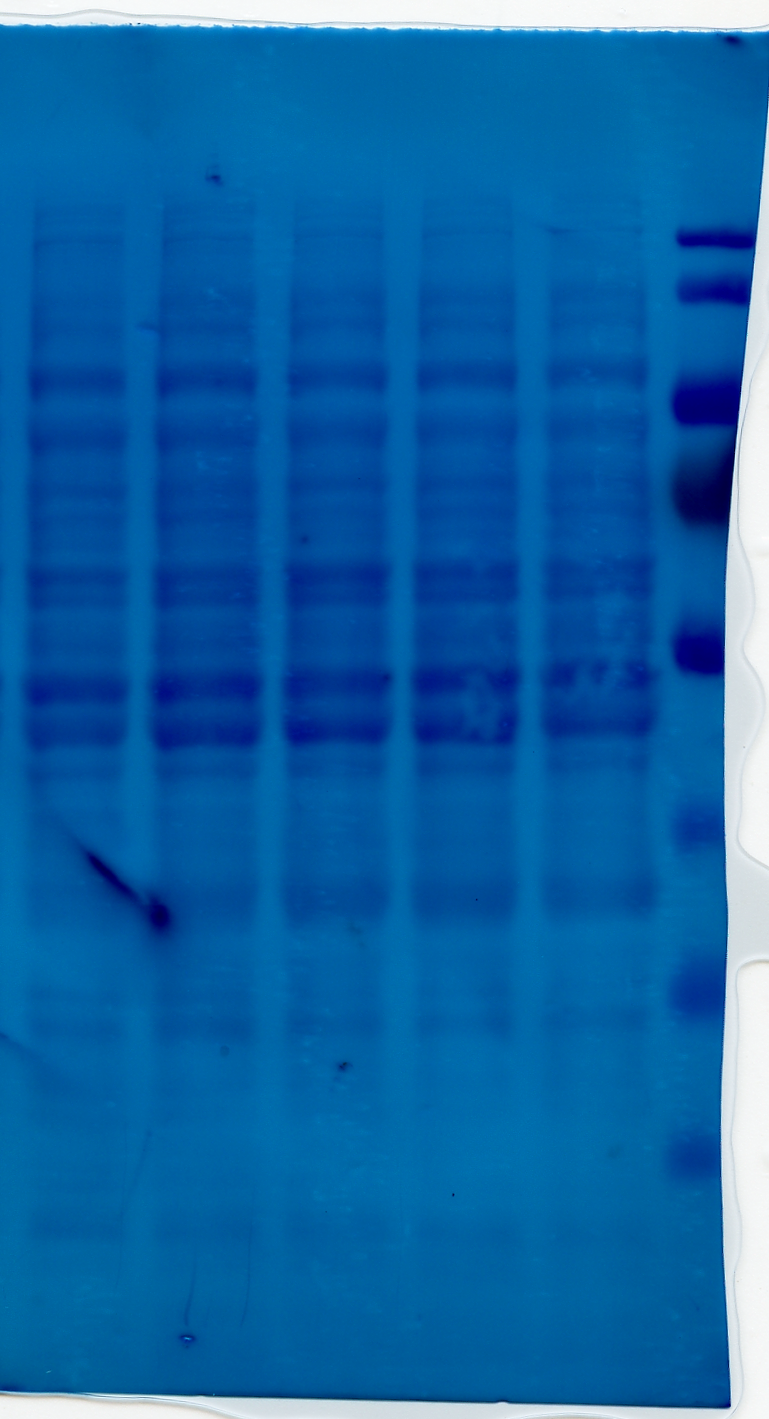

Supplement: Figure 1—figure supplement 1—source data 2. [file elife-85241-fig1-figsupp1-data2.zip › Figure 1-figure supplement 1-source data 2/Figure 1-figure supplement 1-source data 2-2.TIFF]

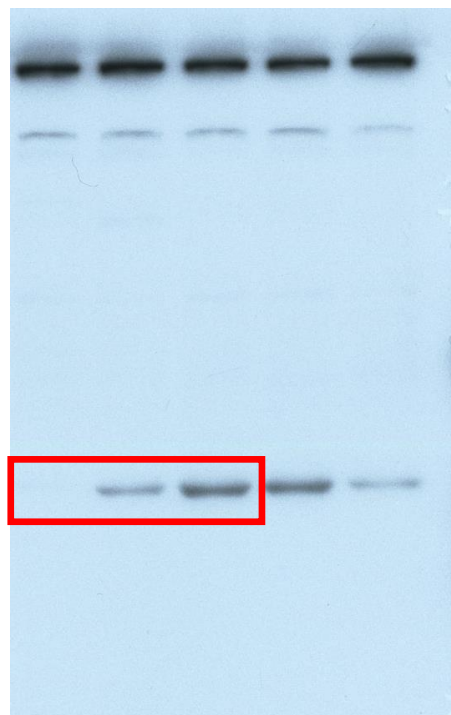

CPC-1

Membrane

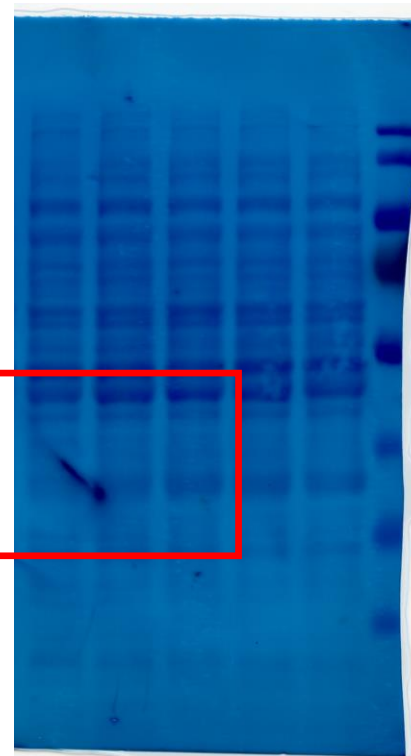

Supplement: Figure 1—figure supplement 1—source data 2. [file elife-85241-fig1-figsupp1-data2.zip › Figure 1-figure supplement 1-source data 2/Figure 1-figure supplement 1-source data 2.pdf]

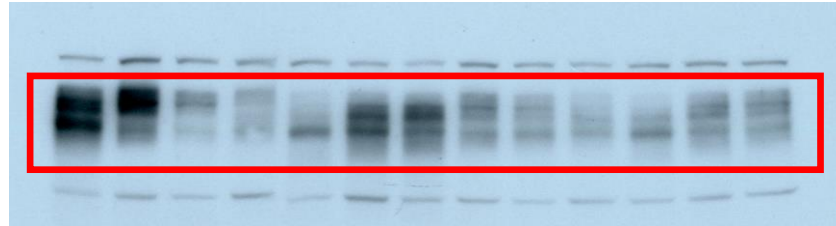

WT

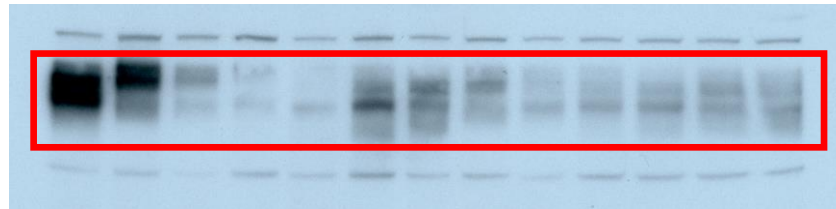

*cpc-3*<sup>ko</sup>

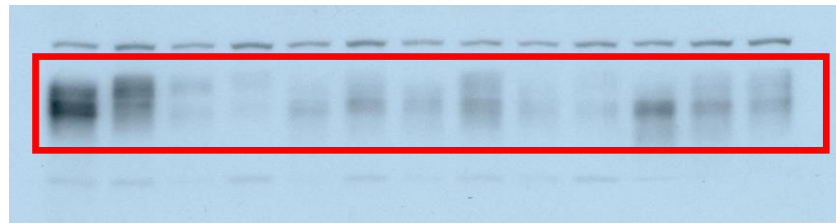

*cpc-1*<sup>ko</sup>

Supplement: Figure 1—figure supplement 2—source data 3. [file elife-85241-fig1-figsupp2-data3.zip › Figure 1-figure supplement 2-source data 3/Figure 1-figure supplement 2-source data 3-1.pdf]

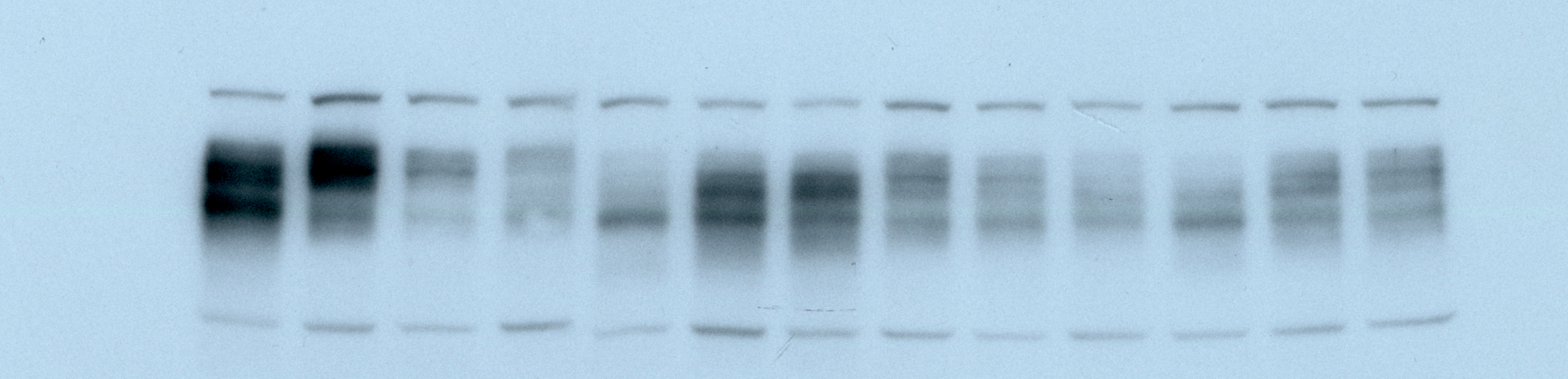

Supplement: Figure 1—figure supplement 2—source data 3. [file elife-85241-fig1-figsupp2-data3.zip › Figure 1-figure supplement 2-source data 3/Figure 1-figure supplement 2-source data 3-3.tif]

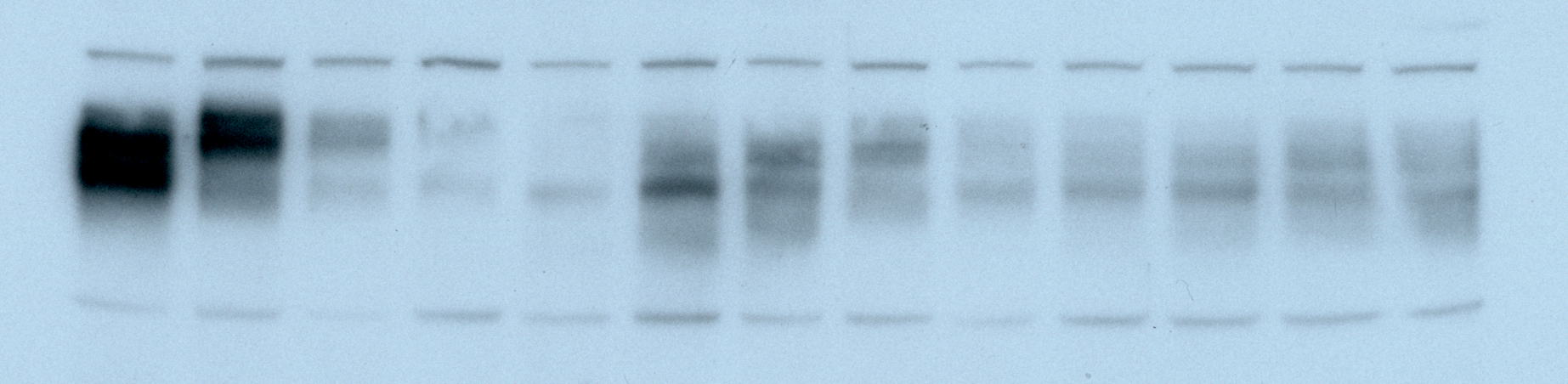

Supplement: Figure 1—figure supplement 2—source data 3. [file elife-85241-fig1-figsupp2-data3.zip › Figure 1-figure supplement 2-source data 3/Figure 1-figure supplement 2-source data 3-4.TIFF]

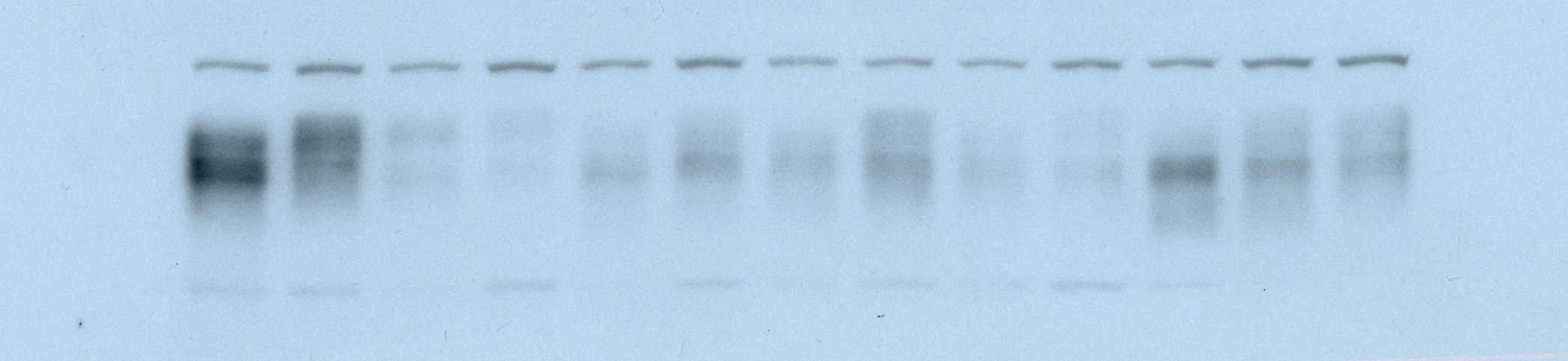

Supplement: Figure 1—figure supplement 2—source data 3. [file elife-85241-fig1-figsupp2-data3.zip › Figure 1-figure supplement 2-source data 3/Figure 1-figure supplement 2-source data 3-5.TIFF]

Northern

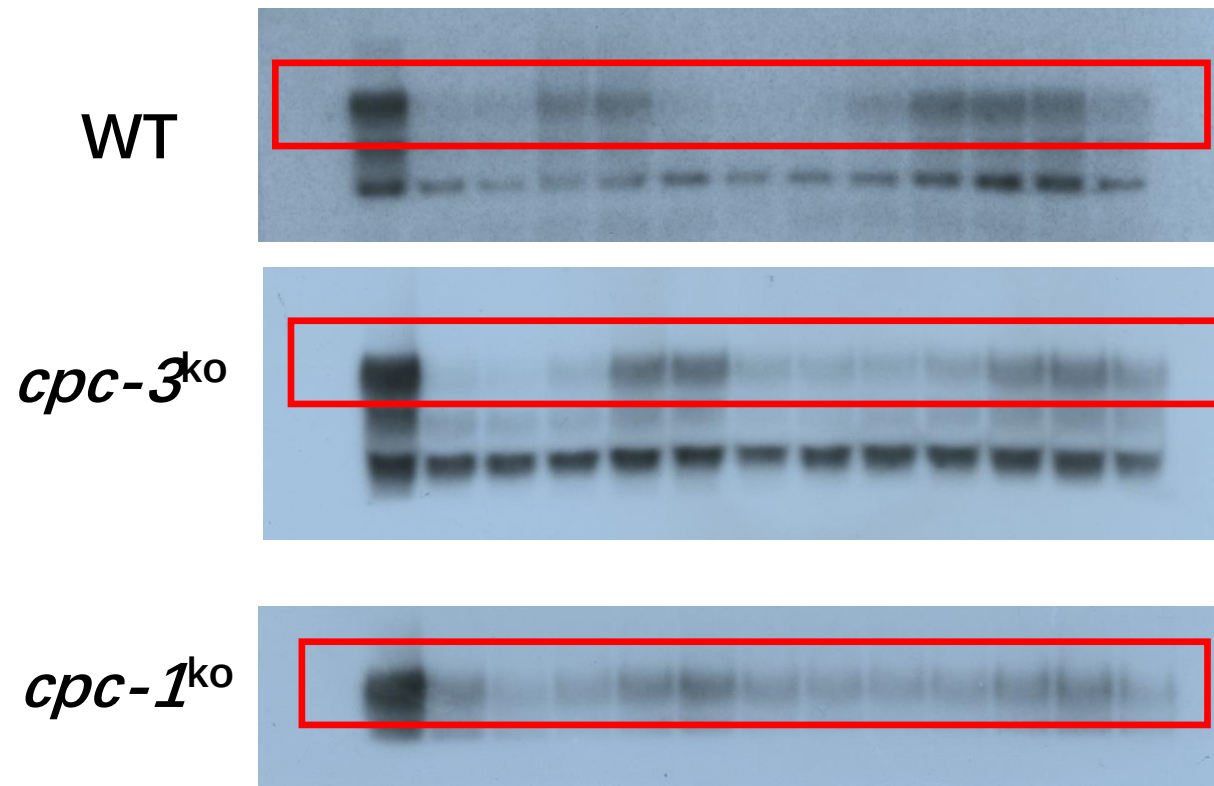

Gel

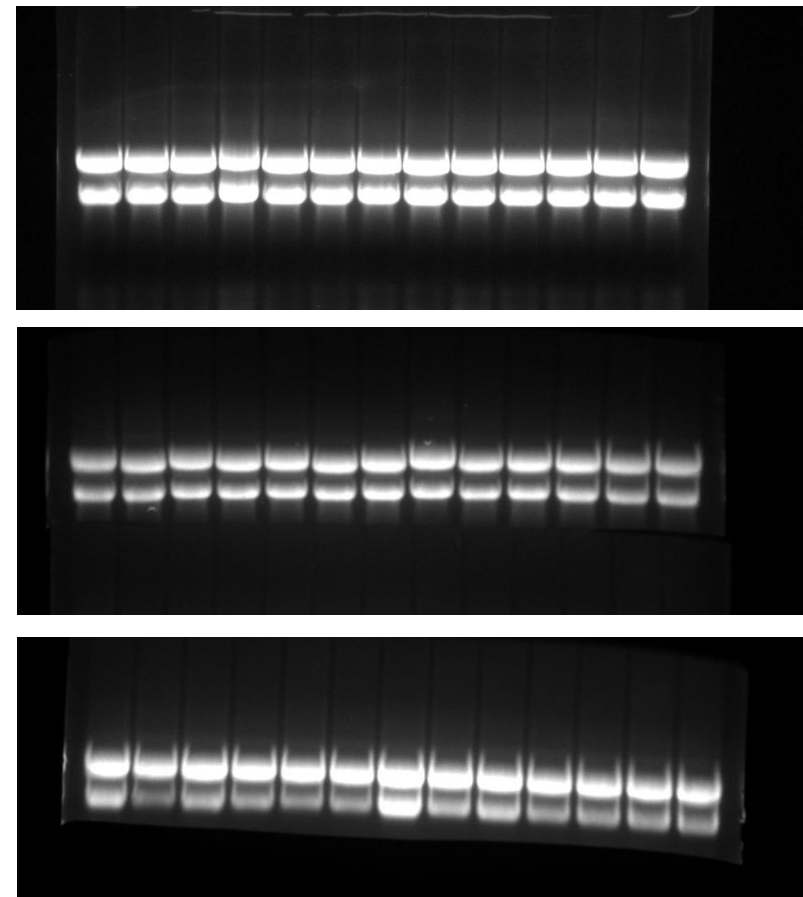

Supplement: Figure 1—figure supplement 2—source data 4. [file elife-85241-fig1-figsupp2-data4.zip › Figure 1-figure supplement 2-source data 4/Figure 1-figure supplement 2-source data 4-1.pdf]

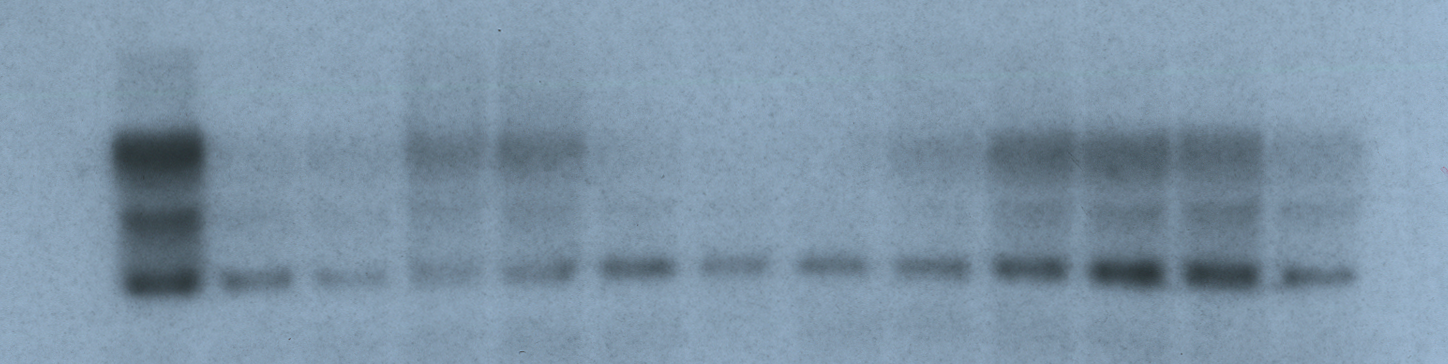

Supplement: Figure 1—figure supplement 2—source data 4. [file elife-85241-fig1-figsupp2-data4.zip › Figure 1-figure supplement 2-source data 4/Figure 1-figure supplement 2-source data 4-3.tif]

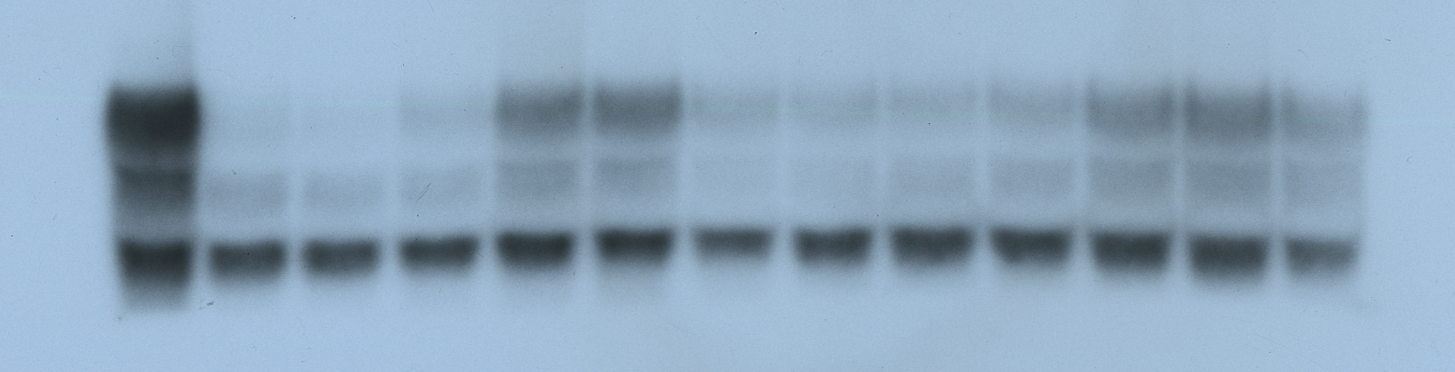

Supplement: Figure 1—figure supplement 2—source data 4. [file elife-85241-fig1-figsupp2-data4.zip › Figure 1-figure supplement 2-source data 4/Figure 1-figure supplement 2-source data 4-4.tif]

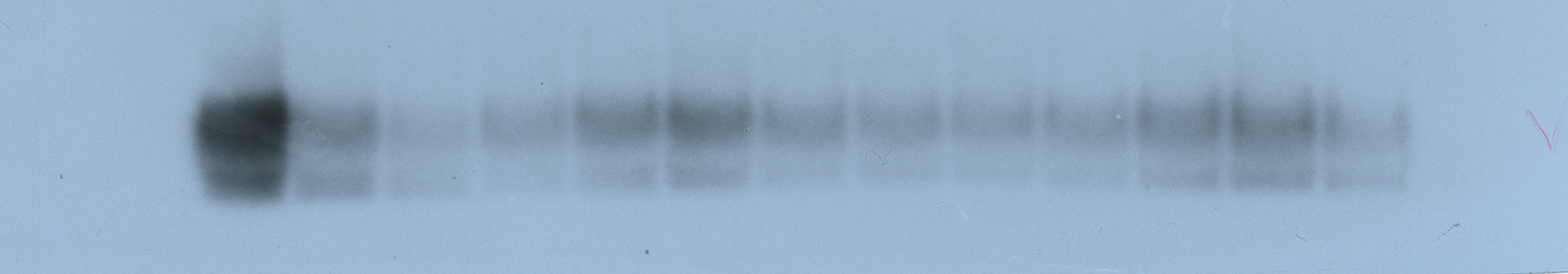

Supplement: Figure 1—figure supplement 2—source data 4. [file elife-85241-fig1-figsupp2-data4.zip › Figure 1-figure supplement 2-source data 4/Figure 1-figure supplement 2-source data 4-5.tif]

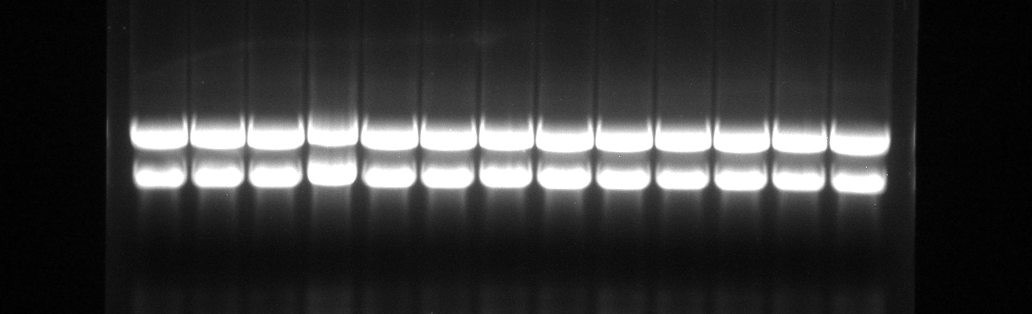

Supplement: Figure 1—figure supplement 2—source data 4. [file elife-85241-fig1-figsupp2-data4.zip › Figure 1-figure supplement 2-source data 4/Figure 1-figure supplement 2-source data 4-6.TIFF]

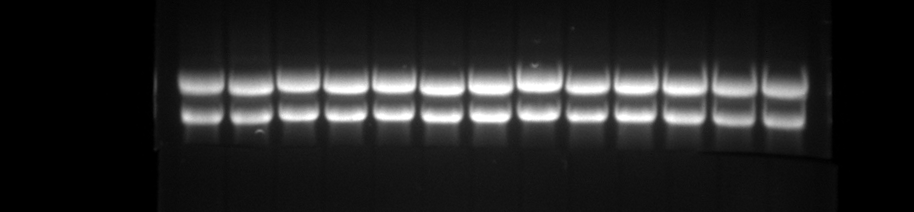

Supplement: Figure 1—figure supplement 2—source data 4. [file elife-85241-fig1-figsupp2-data4.zip › Figure 1-figure supplement 2-source data 4/Figure 1-figure supplement 2-source data 4-7.tif]

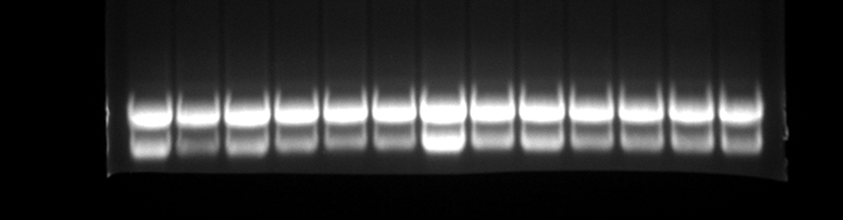

Supplement: Figure 1—figure supplement 2—source data 4. [file elife-85241-fig1-figsupp2-data4.zip › Figure 1-figure supplement 2-source data 4/Figure 1-figure supplement 2-source data 4-8.tif]

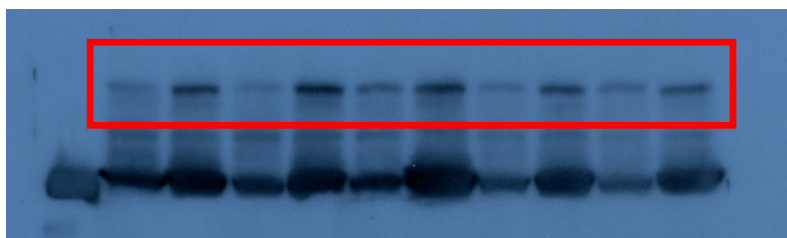

**WC-1**

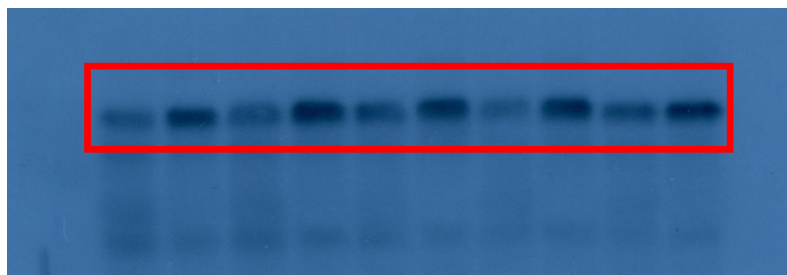

**WC-2**

Supplement: Figure 2—source data 1. [file elife-85241-fig2-data1.zip › Figure 2-source data 1/Figure 2-source data 1-1.pdf]

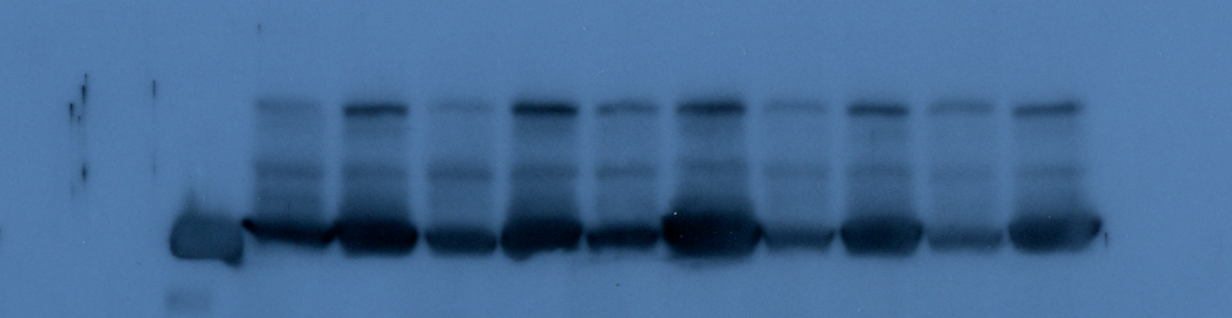

Supplement: Figure 2—source data 1. [file elife-85241-fig2-data1.zip › Figure 2-source data 1/Figure 2-source data 1-2.tif]

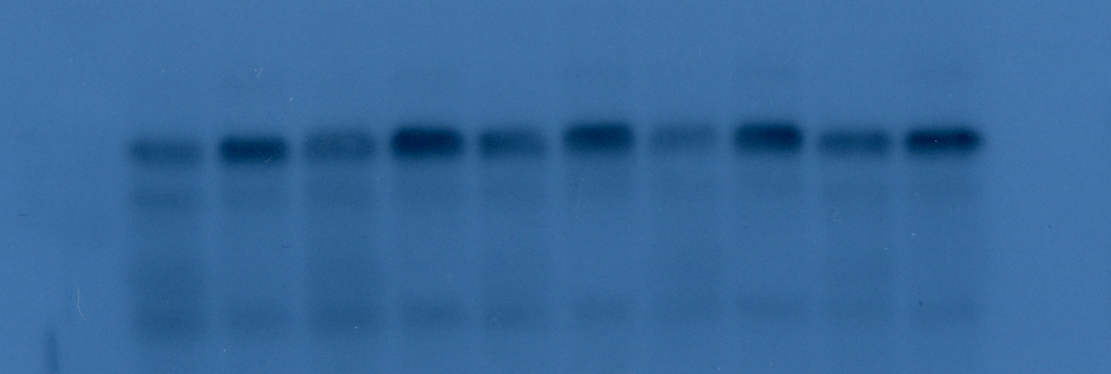

Supplement: Figure 2—source data 1. [file elife-85241-fig2-data1.zip › Figure 2-source data 1/Figure 2-source data 1-3.TIFF]

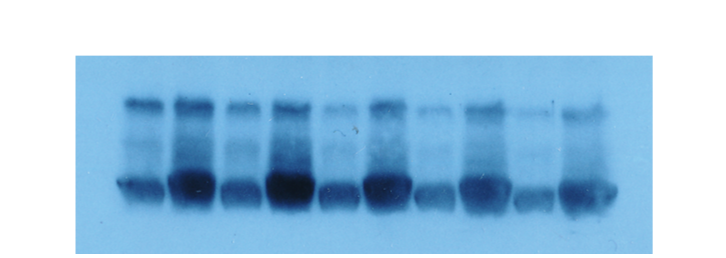

Supplement: Figure 2—source data 2. [file elife-85241-fig2-data2.zip › Figure 2-source data 2/Figure 2-source data 2-1.tif]

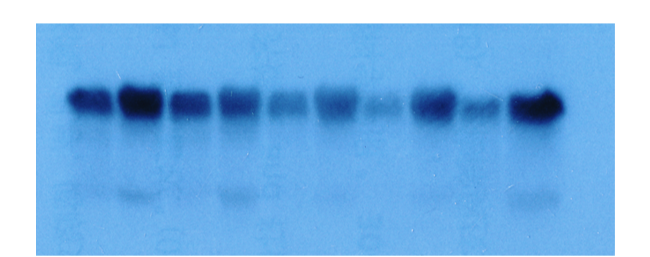

Supplement: Figure 2—source data 2. [file elife-85241-fig2-data2.zip › Figure 2-source data 2/Figure 2-source data 2-2.tif]

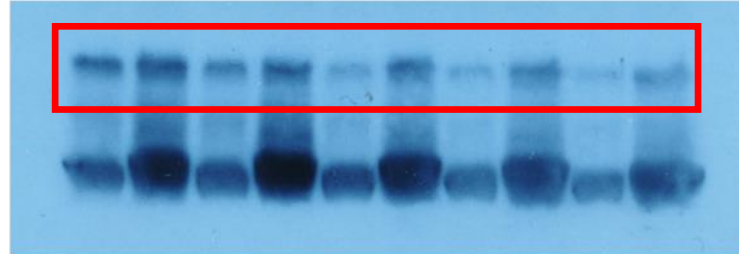

WC-1

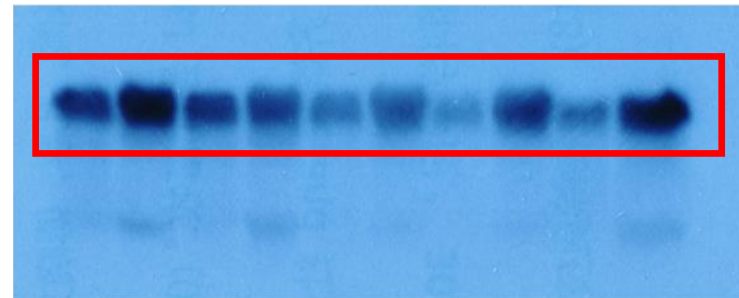

WC-2

Supplement: Figure 2—source data 2. [file elife-85241-fig2-data2.zip › Figure 2-source data 2/Figure 2-source data 2.pdf]

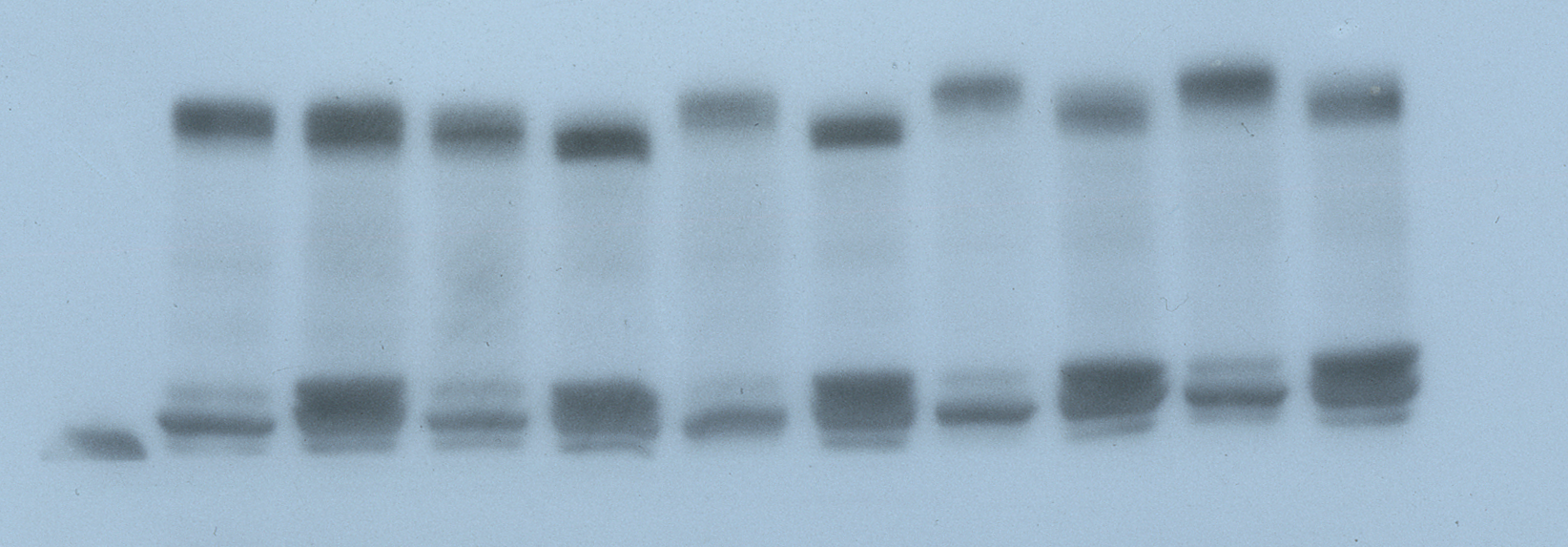

Supplement: Figure 2—figure supplement 1—source data 2. [file elife-85241-fig2-figsupp1-data2.zip › Figure 2-figure supplement 1-source data 2/Figure 2-figure supplement 1-source data 2-1.tif]

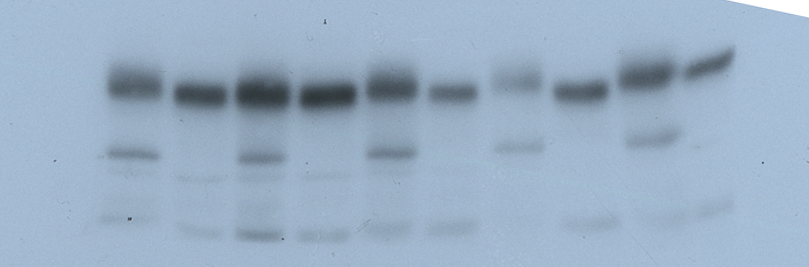

Supplement: Figure 2—figure supplement 1—source data 2. [file elife-85241-fig2-figsupp1-data2.zip › Figure 2-figure supplement 1-source data 2/Figure 2-figure supplement 1-source data 2-2.tif]

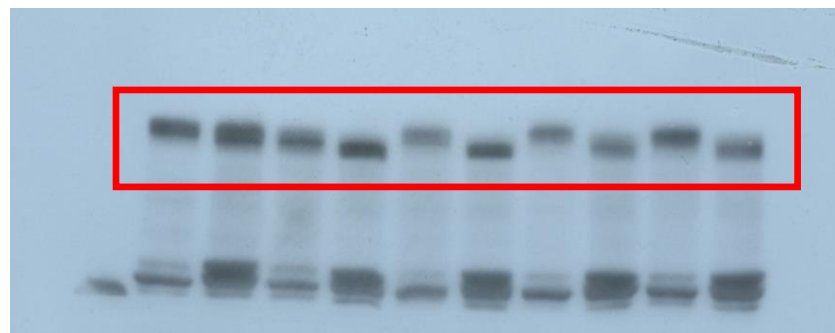

P-WC-1

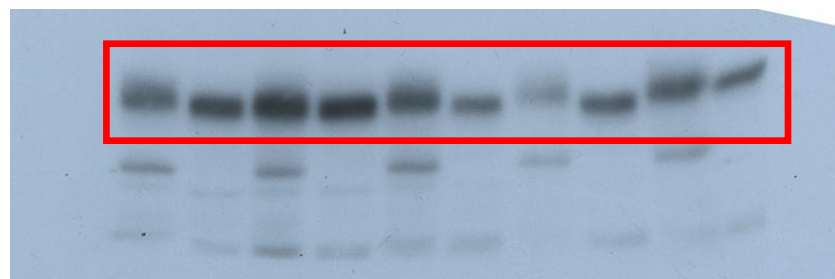

P-WC-2

Supplement: Figure 2—figure supplement 1—source data 2. [file elife-85241-fig2-figsupp1-data2.zip › Figure 2-figure supplement 1-source data 2/Figure 2-figure supplement 1-source data 2.pdf]

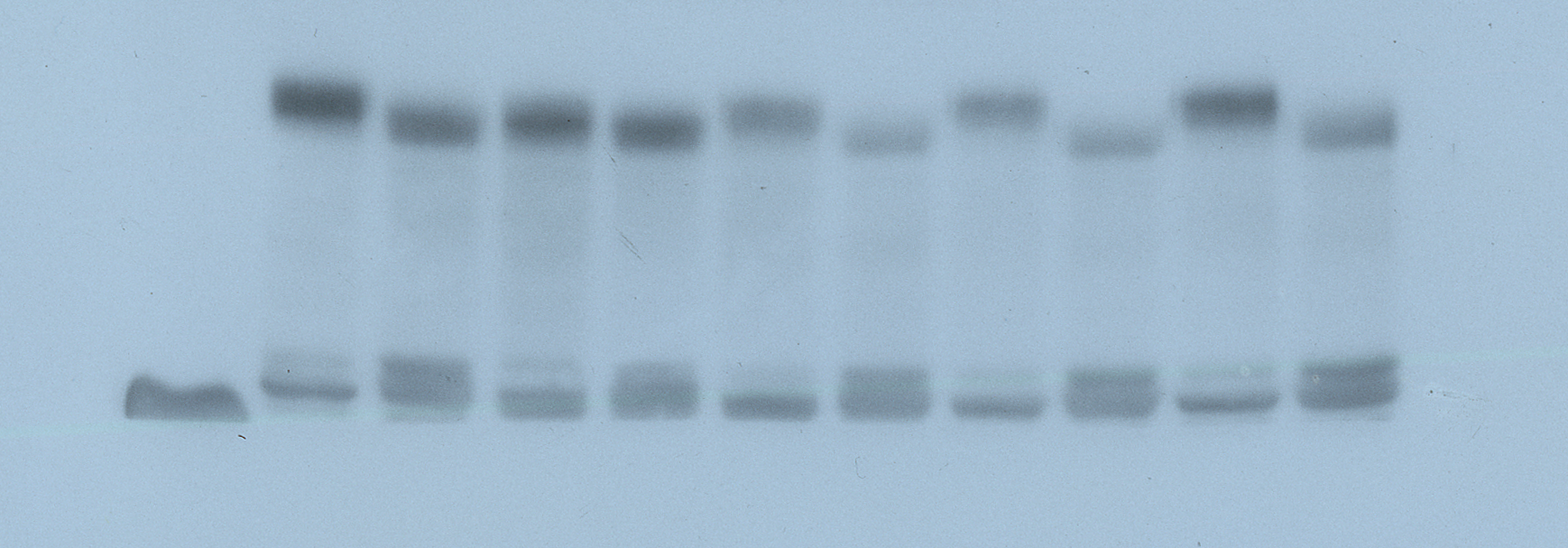

Supplement: Figure 2—figure supplement 1—source data 3. [file elife-85241-fig2-figsupp1-data3.zip › Figure 2-figure supplement 1-source data 3/Figure 2-figure supplement 1-source data 3-1.tif]

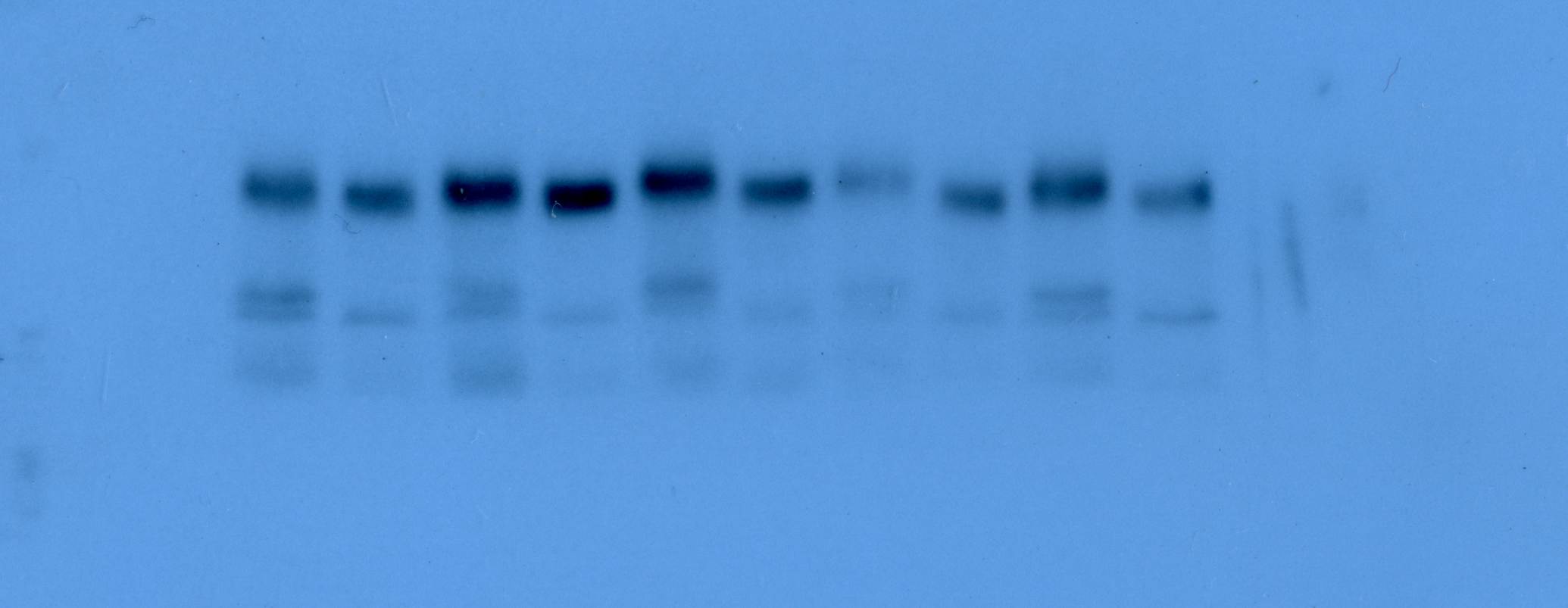

Supplement: Figure 2—figure supplement 1—source data 3. [file elife-85241-fig2-figsupp1-data3.zip › Figure 2-figure supplement 1-source data 3/Figure 2-figure supplement 1-source data 3-2.TIFF]

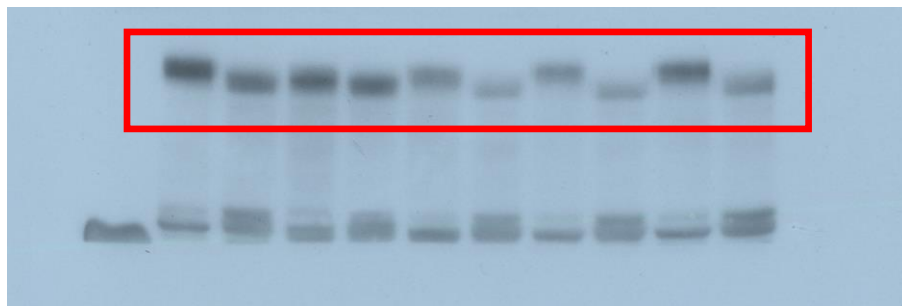

P-WC-1

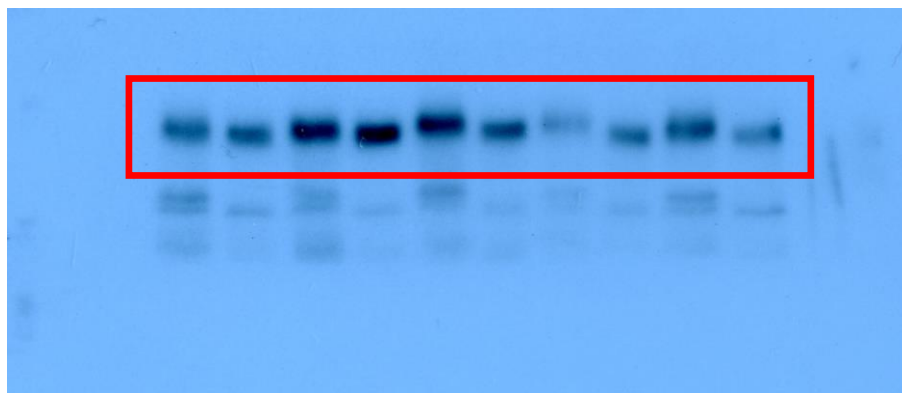

P-WC-2

Supplement: Figure 2—figure supplement 1—source data 3. [file elife-85241-fig2-figsupp1-data3.zip › Figure 2-figure supplement 1-source data 3/Figure 2-figure supplement 1-source data 3.pdf]

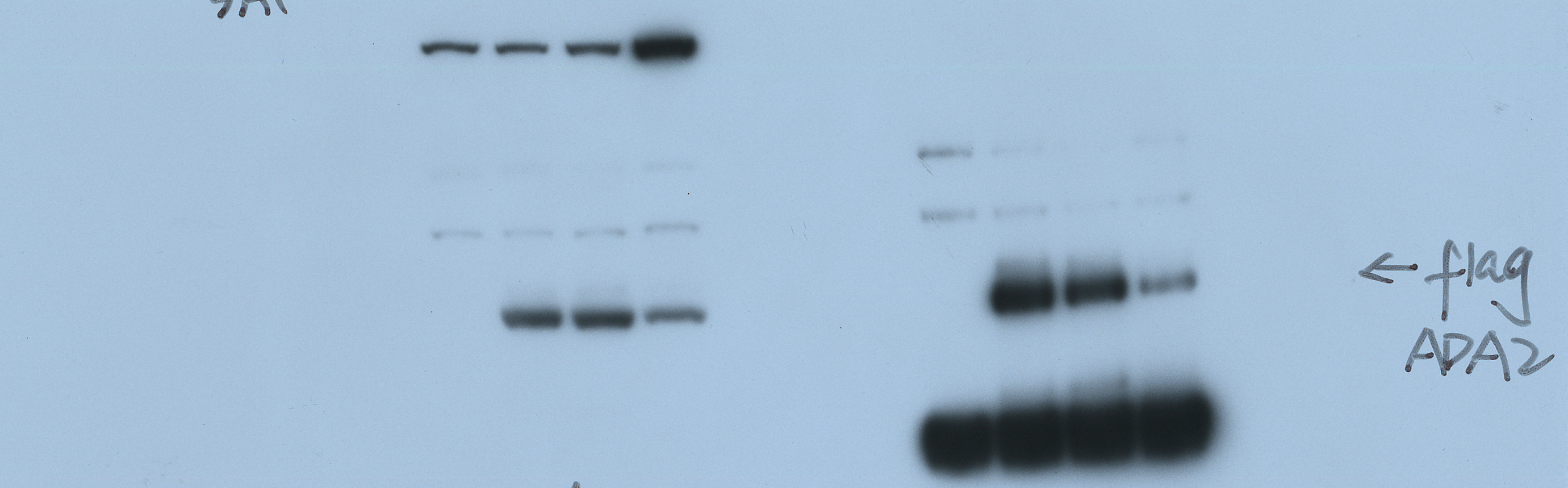

Supplement: Figure 4—source data 2. [file elife-85241-fig4-data2.zip › Figure 4-source data 2/Figure 4-source data 2-1.tif]

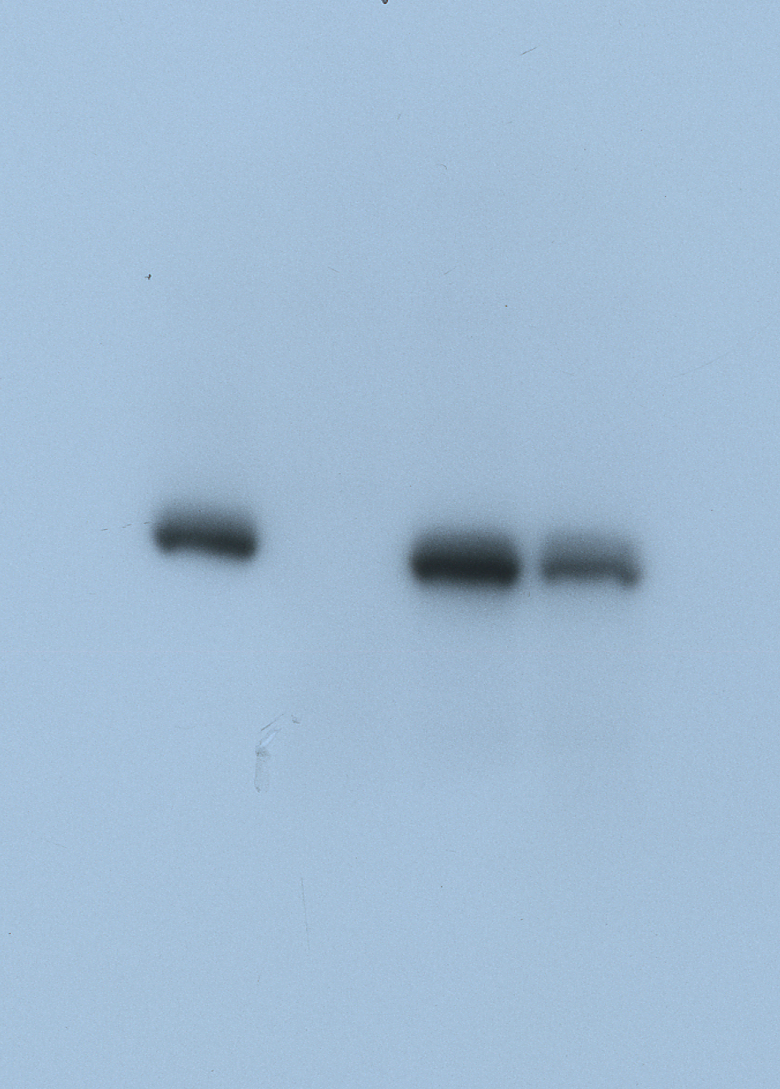

Supplement: Figure 4—source data 2. [file elife-85241-fig4-data2.zip › Figure 4-source data 2/Figure 4-source data 2-2.tif]

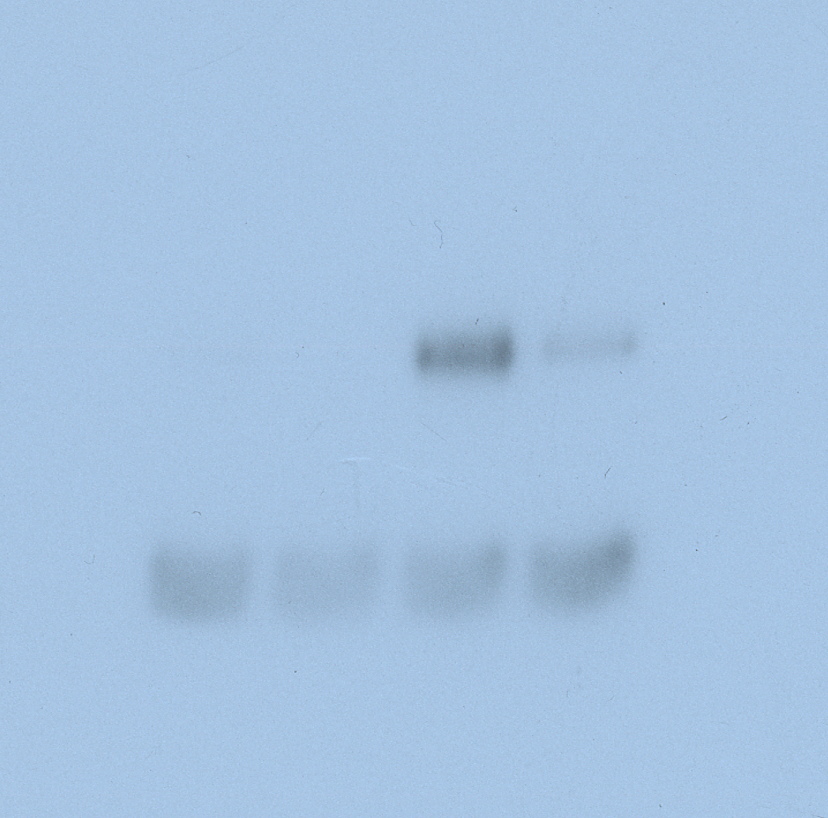

Supplement: Figure 4—source data 2. [file elife-85241-fig4-data2.zip › Figure 4-source data 2/Figure 4-source data 2-3.tif]

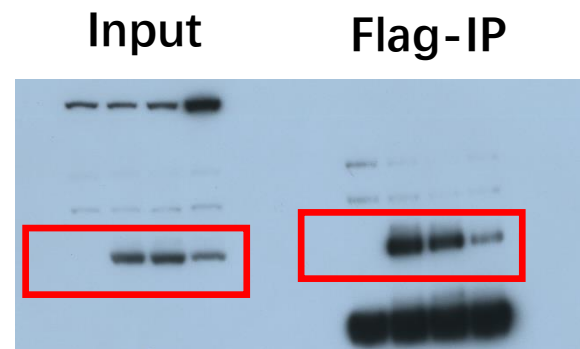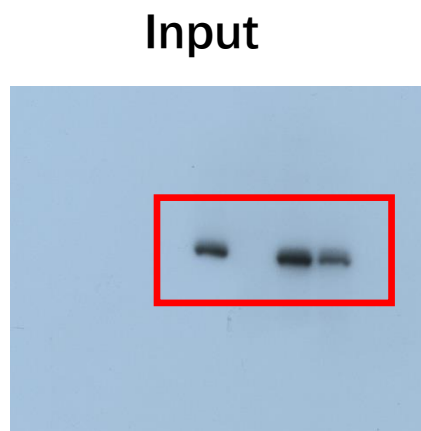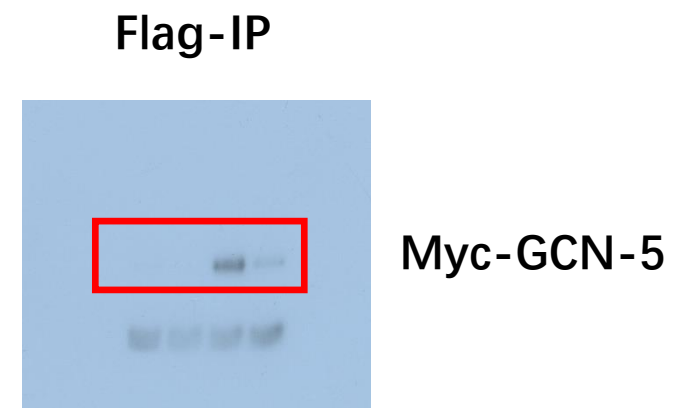

Supplement: Figure 4—source data 2. [file elife-85241-fig4-data2.zip › Figure 4-source data 2/Figure 4-source data 2.pdf]

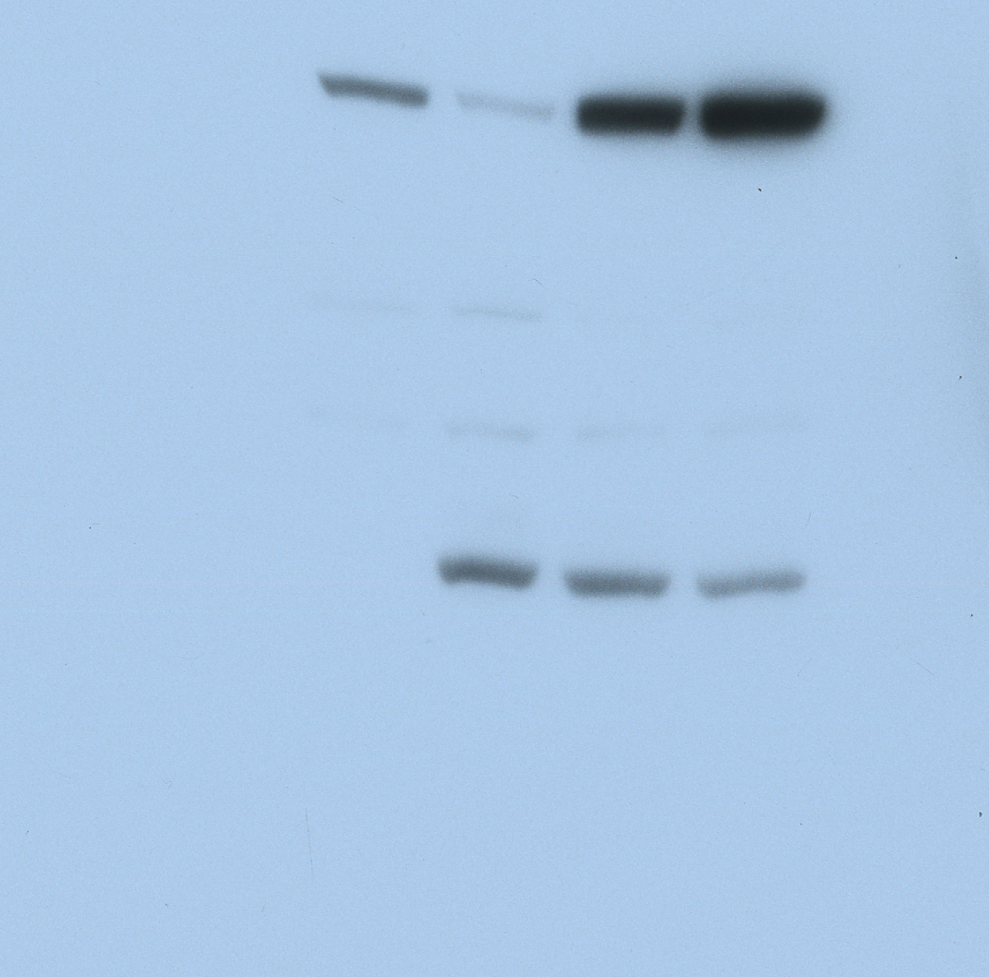

Supplement: Figure 4—source data 3. [file elife-85241-fig4-data3.zip › Figure 4-source data 3/Figure 4-source data 3-1.tif]

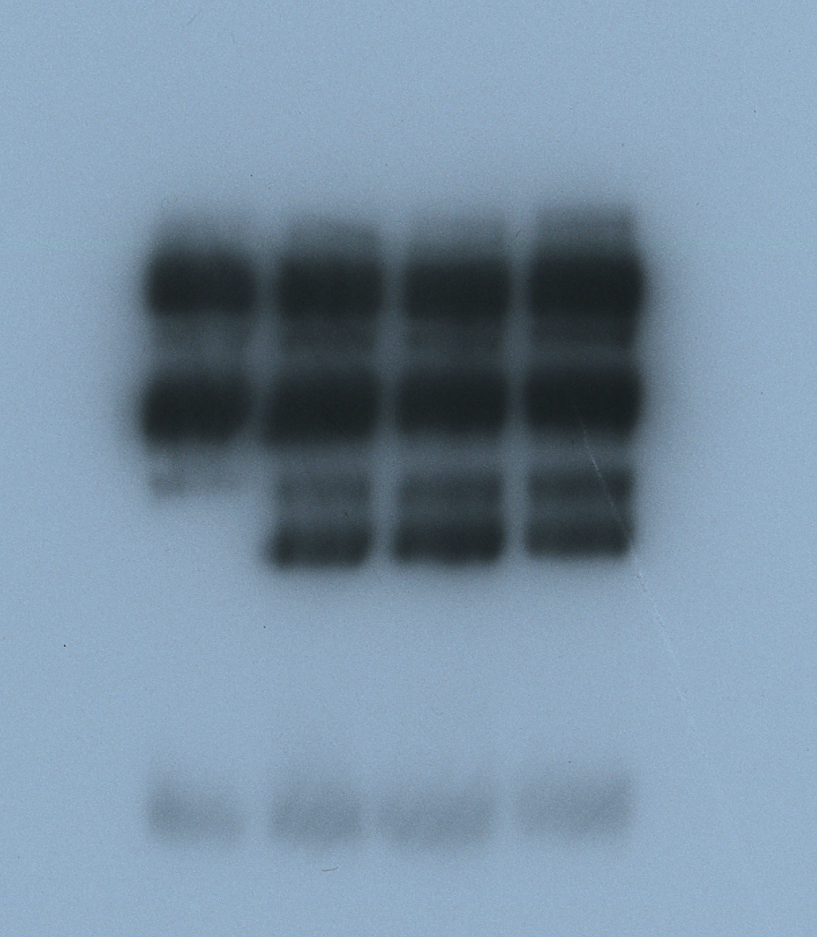

Supplement: Figure 4—source data 3. [file elife-85241-fig4-data3.zip › Figure 4-source data 3/Figure 4-source data 3-2.TIFF]

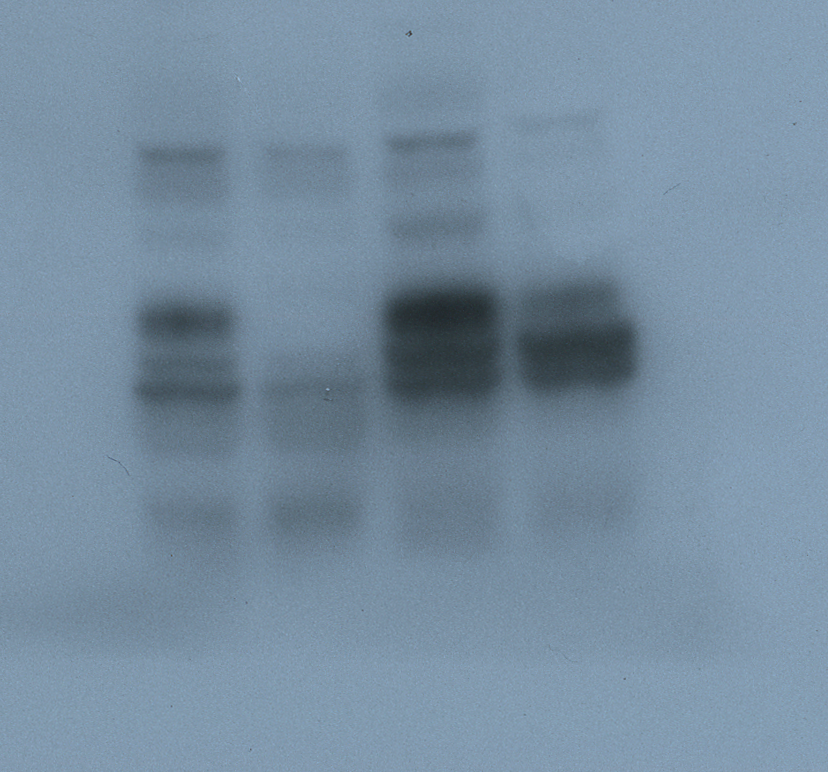

Supplement: Figure 4—source data 3. [file elife-85241-fig4-data3.zip › Figure 4-source data 3/Figure 4-source data 3-3.TIFF]

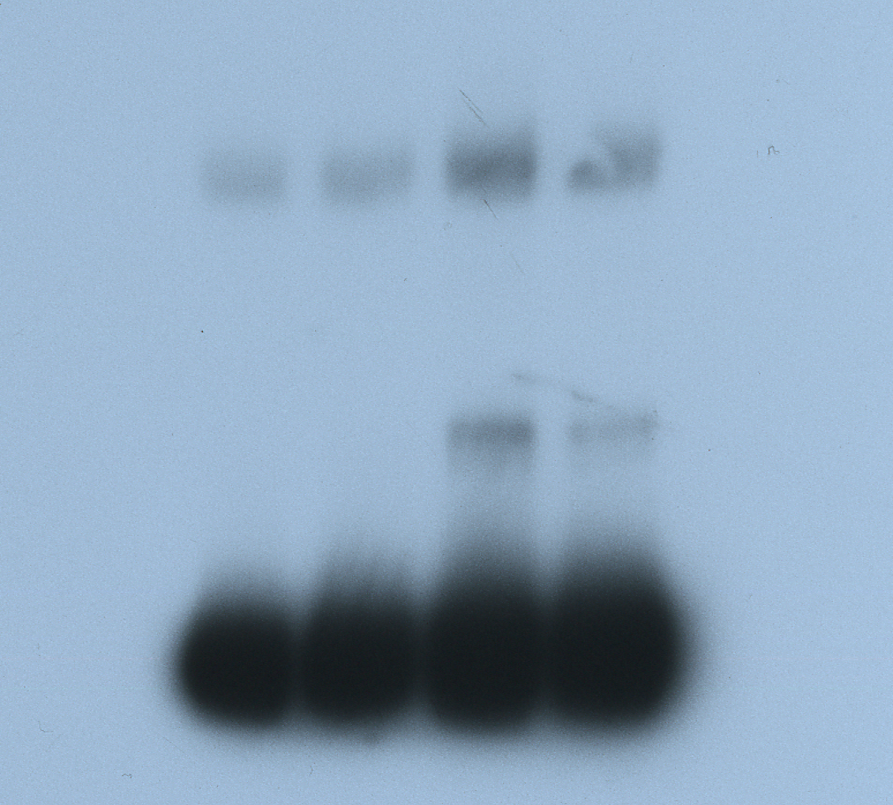

Supplement: Figure 4—source data 3. [file elife-85241-fig4-data3.zip › Figure 4-source data 3/Figure 4-source data 3-4.TIFF]

Input

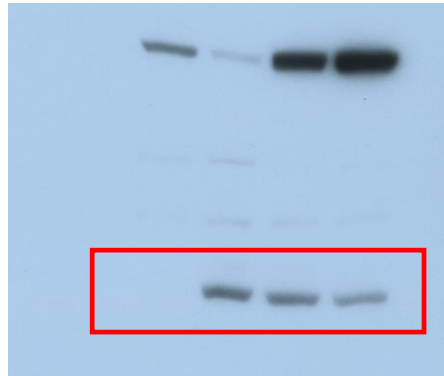

Flag-IP

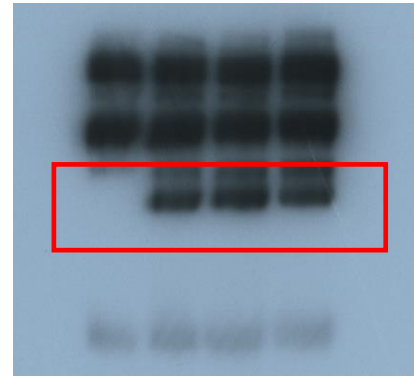

Flag-ADA-2

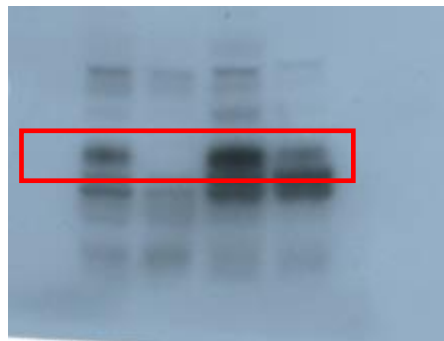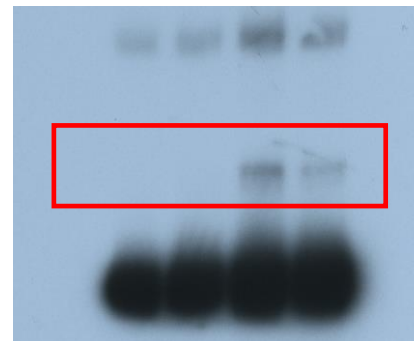

Myc-CPC-1

Supplement: Figure 4—source data 3. [file elife-85241-fig4-data3.zip › Figure 4-source data 3/Figure 4-source data 3.pdf]

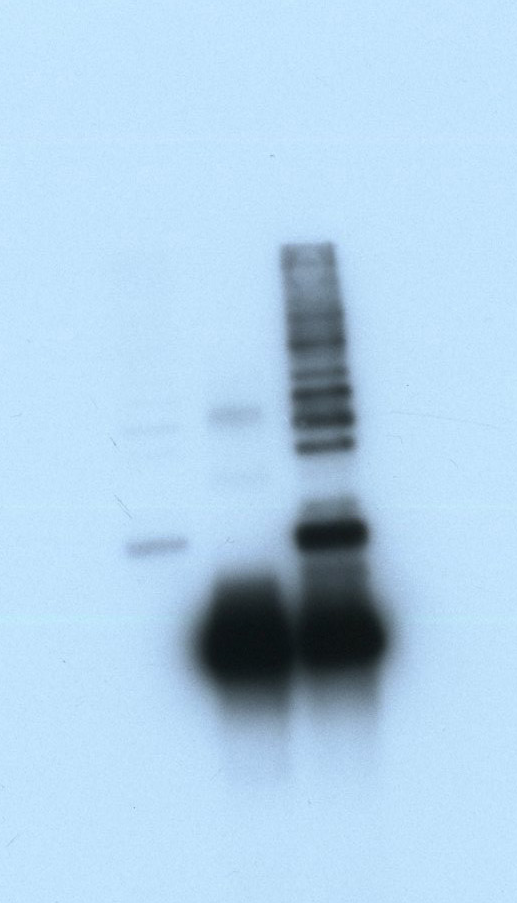

Supplement: Figure 4—figure supplement 1—source data 2. [file elife-85241-fig4-figsupp1-data2.zip › Figure 4-figure supplement 1-source data 2/Figure 4-figure supplement 1-source data 3-1.tif]

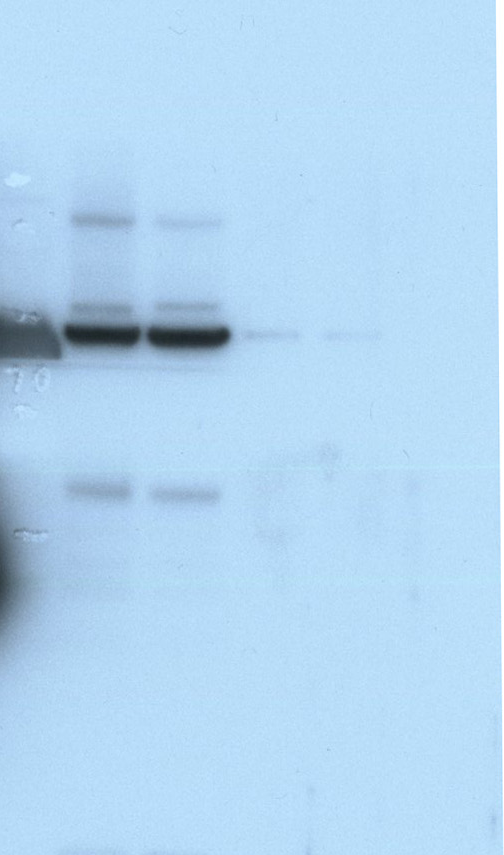

Supplement: Figure 4—figure supplement 1—source data 2. [file elife-85241-fig4-figsupp1-data2.zip › Figure 4-figure supplement 1-source data 2/Figure 4-figure supplement 1-source data 3-2.TIFF]

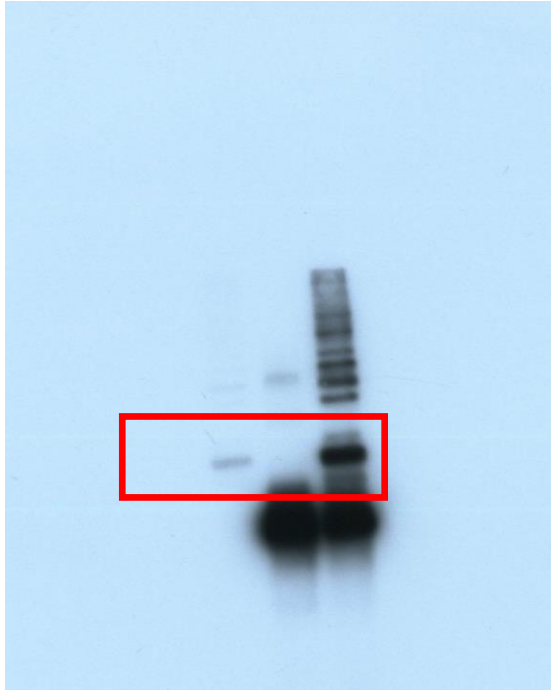

**Myc-CPC-1**

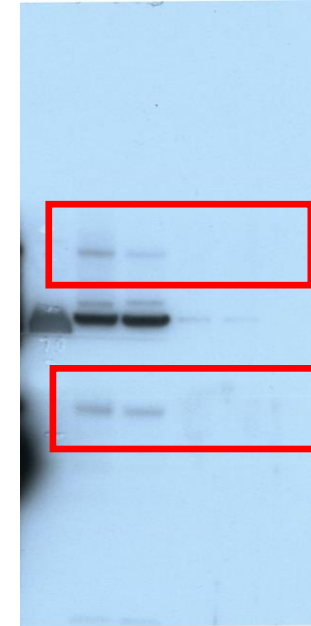

**WC-1**

**WC-2**

Supplement: Figure 4—figure supplement 1—source data 2. [file elife-85241-fig4-figsupp1-data2.zip › Figure 4-figure supplement 1-source data 2/Figure 4-figure supplement 1-source data 3.pdf]

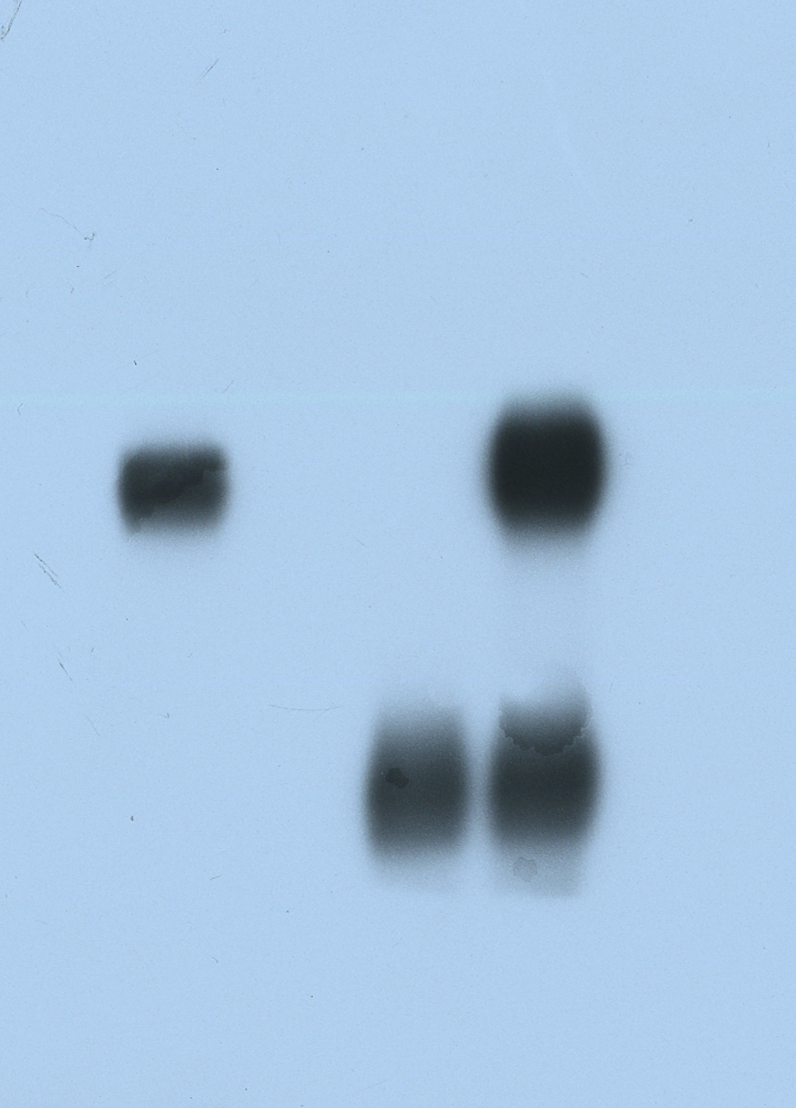

Supplement: Figure 4—figure supplement 1—source data 3. [file elife-85241-fig4-figsupp1-data3.zip › Figure 4-figure supplement 1-source data 3/Figure 4-figure supplement 1-source data 3-1.tif]

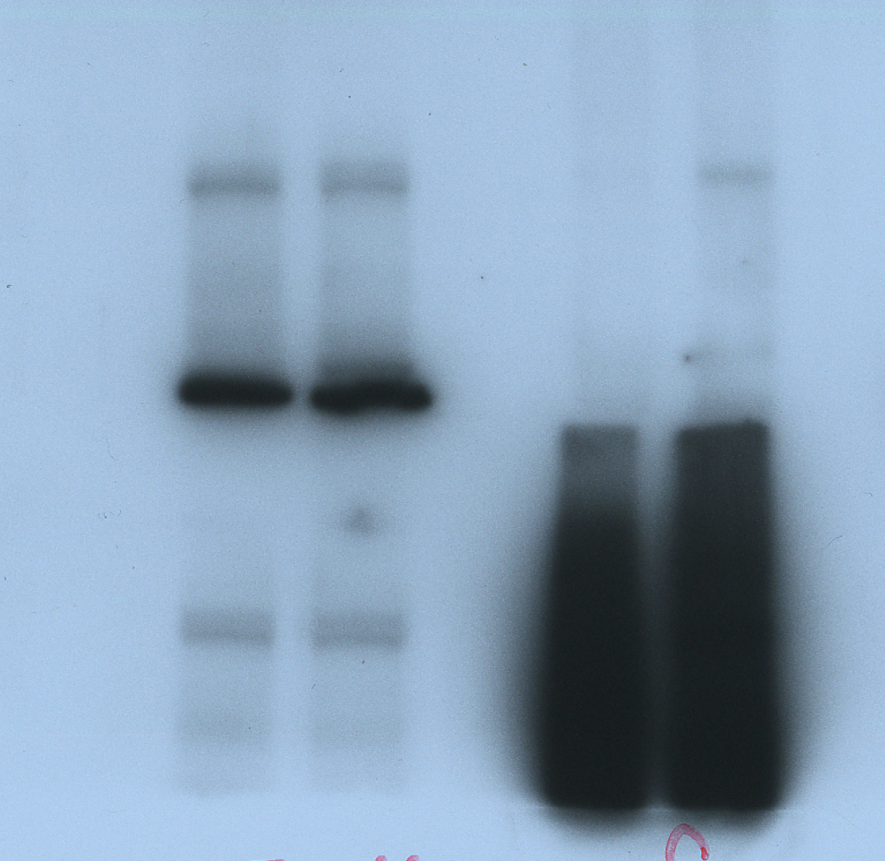

Supplement: Figure 4—figure supplement 1—source data 3. [file elife-85241-fig4-figsupp1-data3.zip › Figure 4-figure supplement 1-source data 3/Figure 4-figure supplement 1-source data 3-2.TIFF]

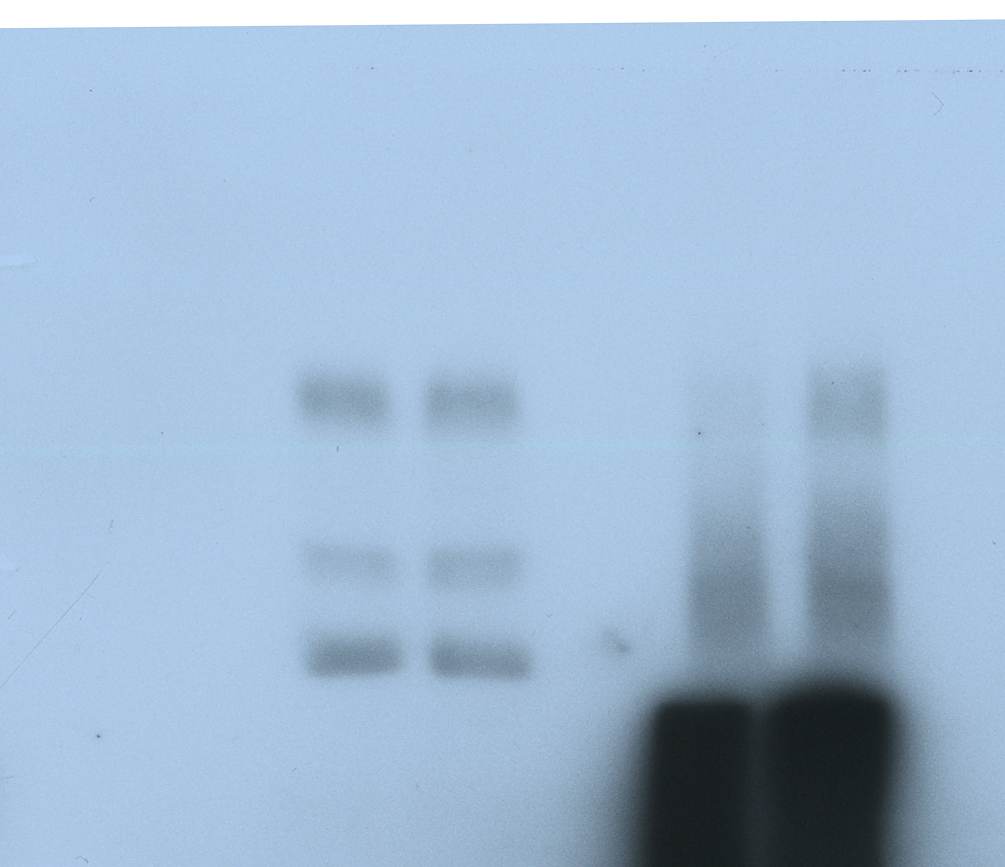

Supplement: Figure 4—figure supplement 1—source data 3. [file elife-85241-fig4-figsupp1-data3.zip › Figure 4-figure supplement 1-source data 3/Figure 4-figure supplement 1-source data 3-3.TIFF]

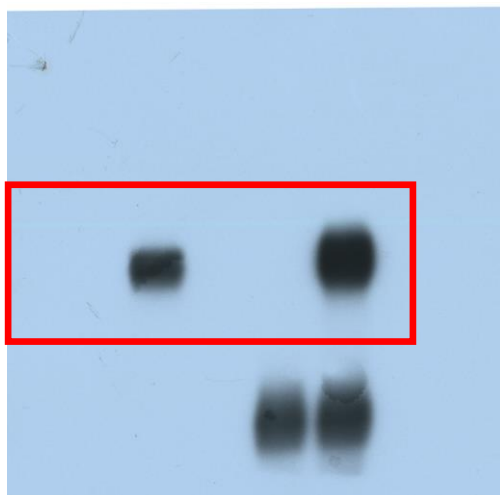

Myc-GCN-5

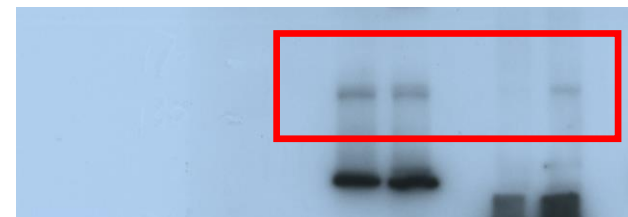

WC-1

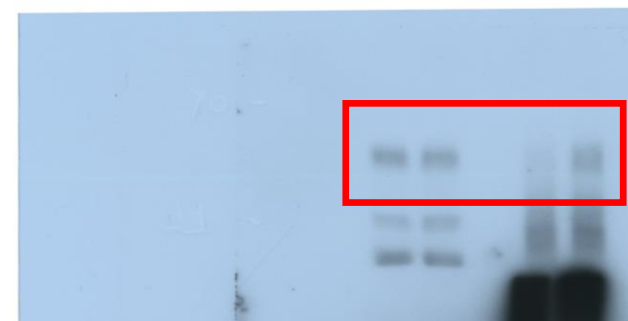

WC-2

Supplement: Figure 4—figure supplement 1—source data 3. [file elife-85241-fig4-figsupp1-data3.zip › Figure 4-figure supplement 1-source data 3/Figure 4-figure supplement 1-source data 3.pdf]

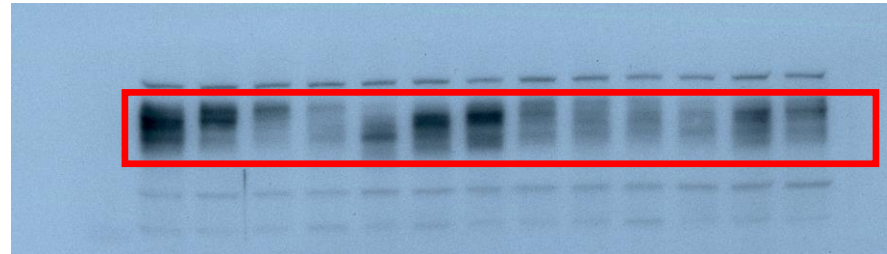

WT

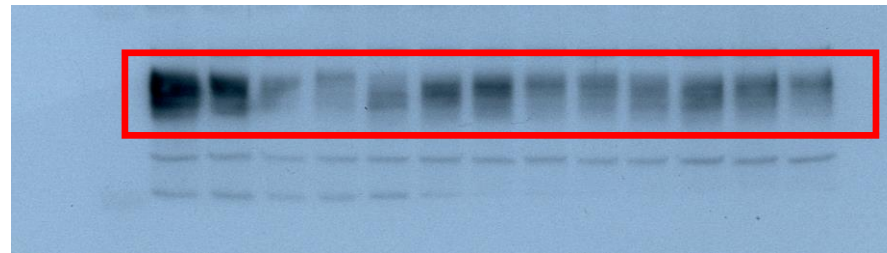

*gcn-5*<sup>ko</sup>

Supplement: Figure 5—source data 2. [file elife-85241-fig5-data2.zip › Figure 5-source data 2/Figure 5-source data 2-1.pdf]

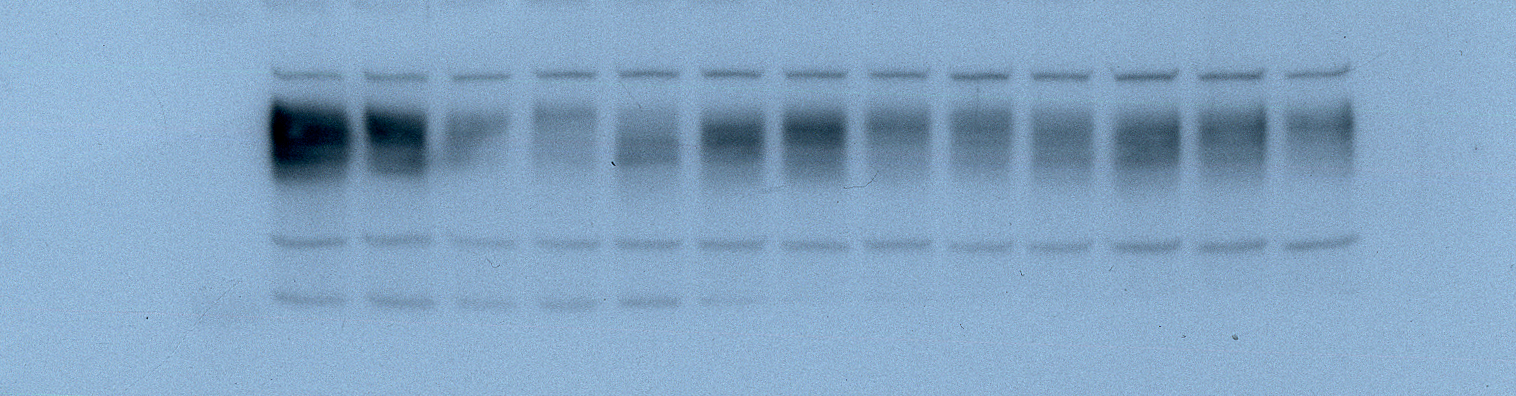

Supplement: Figure 5—source data 2. [file elife-85241-fig5-data2.zip › Figure 5-source data 2/Figure 5-source data 2-2.TIFF]

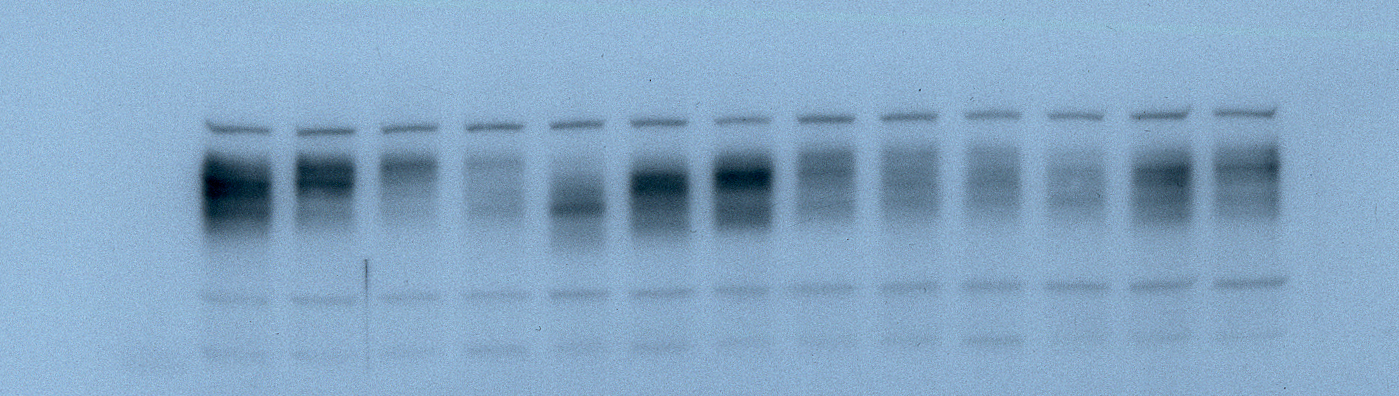

Supplement: Figure 5—source data 2. [file elife-85241-fig5-data2.zip › Figure 5-source data 2/Figure 5-source data 2-3.tif]

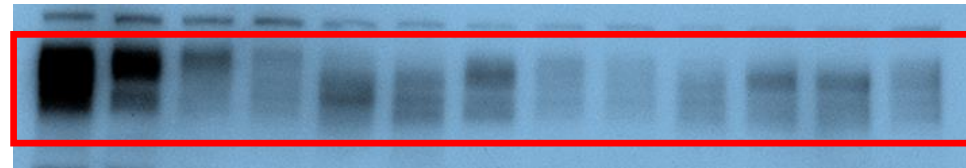

WT

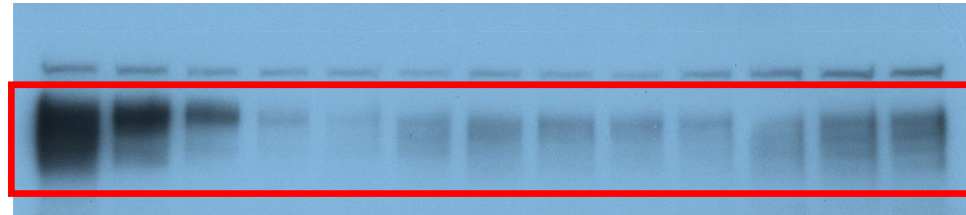

*ada-2*<sup>ko</sup>

Supplement: Figure 5—figure supplement 1—source data 3. [file elife-85241-fig5-figsupp1-data3.zip › Figure 5-figure supplement 1-source data 3/Figure 5-figure supplement 1-source data 3-1.pdf]

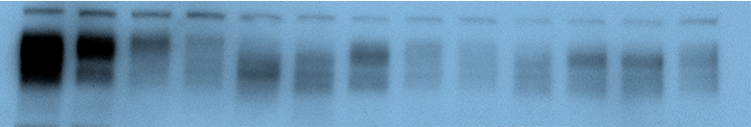

Supplement: Figure 5—figure supplement 1—source data 3. [file elife-85241-fig5-figsupp1-data3.zip › Figure 5-figure supplement 1-source data 3/Figure 5-figure supplement 1-source data 3-2.tif]

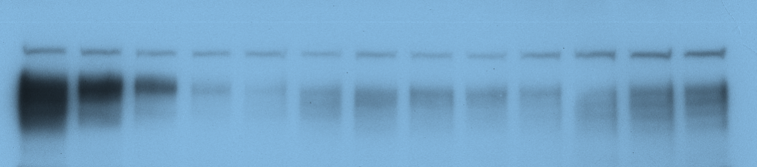

Supplement: Figure 5—figure supplement 1—source data 3. [file elife-85241-fig5-figsupp1-data3.zip › Figure 5-figure supplement 1-source data 3/Figure 5-figure supplement 1-source data 3-3.tif]
